# Cost‐effectiveness of integrated treatment for hepatitis C virus (HCV) among people who inject drugs in Norway: An economic evaluation of the INTRO‐HCV trial

**DOI:** 10.1111/add.16305

**Published:** 2023-07-29

**Authors:** Aaron Guanliang Lim, Christer Frode Aas, Ege Su Çağlar, Jørn Henrik Vold, Lars Thore Fadnes, Peter Vickerman, Kjell Arne Johansson

**Affiliations:** ^1^ Population Health Sciences, Bristol Medical School University of Bristol Bristol UK; ^2^ Bergen Addiction Research, Department of Addiction Medicine Haukeland University Hospital Bergen Norway; ^3^ Department of Global Public Health and Primary Care University of Bergen Bergen Norway; ^4^ Division of Psychiatry Haukeland University Hospital Bergen Norway

**Keywords:** direct‐acting antivirals, health‐related quality of life, Markov model, opiate substitution treatment, opioid agonist therapy, willingness‐to‐pay

## Abstract

**Background and aims:**

The INTRO‐HCV randomized controlled trial conducted in Norway over 2017–2019 found that integrated treatment, compared with standard‐of‐care hospital treatment, for hepatitis C virus (HCV) with direct‐acting antivirals (DAAs) improved treatment outcomes among people who inject drugs (PWID). We evaluated cost‐effectiveness of the INTRO‐HCV intervention.

**Design:**

A Markov health state transition model of HCV disease progression and treatment with cost‐effectiveness analysis from the health‐provider perspective. Primary cost, utility, and health outcome data were derived from the trial. Costs and health benefits (quality‐adjusted life‐years, QALYs) were tracked over 50 years. Probabilistic and univariate sensitivity analyses investigated DAA price reductions and variations in HCV treatment and disease care cost assumptions, using costs from different countries (Norway, United Kingdom, United States, France, Australia).

**Setting and participants:**

PWID attending community‐based drug treatment centers for people with opioid dependence in Norway.

**Measurements:**

Incremental cost‐effectiveness ratio (ICER) in terms of cost per QALY gained, compared against a conventional (€70 000/QALY) willingness‐to‐pay threshold for Norway and lower (€20 000/QALY) threshold common among high‐income countries.

**Findings:**

Integrated treatment resulted in an ICER of €13 300/QALY gained, with 99% and 71% probability of being cost‐effective against conventional and lower willingness‐to‐pay thresholds, respectively. A 30% lower DAA price reduced the ICER to €6 900/QALY gained, with 91% probability of being cost‐effective at the lower willingness‐to‐pay threshold. A 60% and 90% lower DAA price had 36% and >99% probability of being cost‐saving, respectively. Sensitivity analyses suggest integrated treatment was cost‐effective at the lower willingness‐to‐pay threshold (>60% probability) across different assumptions on HCV treatment and disease care costs with 30% DAA price reduction, and became cost‐saving with 60%–90% price reductions.

**Conclusions:**

Integrated hepatitis C virus treatment for people who inject drugs in community settings is likely cost‐effective compared with standard‐of‐care referral pathways in Norway and may be cost‐saving in settings with particular characteristics.

## INTRODUCTION

Injecting drug use (IDU) is a key driver of hepatitis C virus (HCV) transmission [1, 2]. Despite the development of highly efficacious direct‐acting antiviral (DAA) treatments for HCV, people who inject drugs (PWID) frequently have low levels of diagnosis and treatment because of stigma and lack of access to services [3]. Implementing HCV treatment through efficient and integrated delivery platforms can help overcome these barriers by increasing accessibility to DAAs among PWID, which is urgently required to progress toward achieving World Health Organization (WHO) targets for eliminating HCV globally [4]. However, despite considerable potential for cost‐savings, until recently evidence supporting integrated care for HCV among PWID has been limited [5].

The integrated treatment of hepatitis C (INTRO‐HCV) study [6] was a multi‐center, randomized controlled trial that enrolled 298 HCV‐infected PWID attending one of eight multidisciplinary opioid agonist therapy (OAT) clinics or two community care centers (CCC) in Norway over 2017 to 2019. The purpose of the trial was to evaluate the impact on HCV treatment outcomes of integrated HCV treatment within decentralized OAT clinics and CCC compared to standard‐of‐care treatment occurring in hospital outpatient clinics.

Overall, the INTRO‐HCV trial demonstrated that the integrated pathway led to superior treatment outcomes in terms of a higher proportion of patients initiating treatment (integrated, 98% vs standard‐of‐care, 77%), a faster time to treatment initiation (hazard ratio of 2.2), and a higher proportion achieving sustained virological response (SVR) (effective cure) following treatment (integrated, 95%; standard‐of‐care, 88% among those completed treated and polymerase chain reaction [PCR]‐tested post‐treatment) [7].

In this study, we use modelling combined with the INTRO‐HCV trial findings [7] to estimate the long‐term costs, health outcomes and cost‐effectiveness of the integrated HCV treatment delivery approach versus the standard‐of‐care treatment referral to hospital outpatient clinics for PWID in community settings.

## METHODS

### Study description

The INTRO‐HCV trial recruited PWID from OAT clinics or CCC in Bergen and Stavanger (Western Norway) who were diagnosed with detectable HCV RNA and eligible for HCV treatment. Randomization occurred after recruitment. DAA regimens were used in both treatment pathways, with the primary trial outcomes being treatment uptake and SVR assessed at 12 weeks after treatment completion.

The integrated treatment pathway involved HCV treatment being delivered at OAT clinics or CCC by on‐site multidisciplinary teams, including clinical specialists in addiction medicine, psychologists providing mental health services, nurses, social workers and peer counsellors. Patients had frequent follow‐up appointments (usually several times per week) for substance use disorders or other medical conditions, with follow‐up related to HCV treatment being integrated into these appointments.

The standard‐of‐care treatment pathway involved patients being referred for clinical assessment, HCV treatment and 4 to 5 follow‐up consultations at a standard medical outpatient hospital clinic within 1 to 25 km travelling distance, with transportation costs being incurred by the patient. Appointments unrelated to HCV were scheduled separately.

### Model description

**FIGURE 1:**
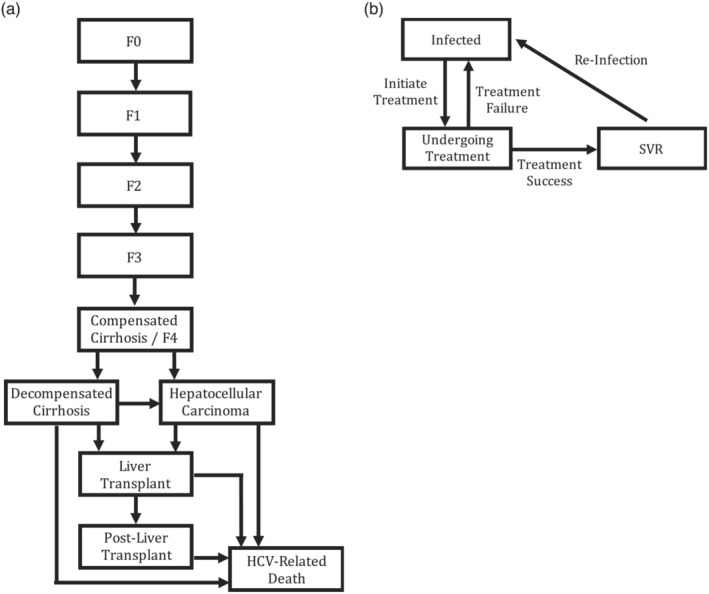
Schematic of Markov model showing stratification by (a) untreated chronic HCV disease progression and liver disease states, and (b) HCV infection, treatment or SVR status. Achieving SVR (i.e. cure) results in further liver disease progression being halted for those in METAVIR stages F0–F3, or reduced for those with CC, DC or HCC. CC, compensated cirrhosis, equivalent to METAVIR stage F4; DC, decompensated cirrhosis; HCC, hepatocellular carcinoma; HCV, hepatitis C virus; METAVIR, meta‐analysis of histological data in viral hepatitis; SVR, sustained virological response.

We developed a closed cohort Markov model of HCV disease progression and treatment among HCV‐diagnosed individuals (Figure 1), based on the INTRO‐HCV cohort. The model was stratified by HCV‐associated disease progression (Figure 1a), with two mild fibrosis states (meta‐analysis of histological data in viral hepatitis [METAVIR] stages F0–F1), two moderate fibrosis states (METAVIR stages F2–F3), compensated cirrhosis (CC) (METAVIR stage F4), decompensated cirrhosis (DC), hepatocellular carcinoma (HCC), liver transplantation (LT) and post‐liver transplantation (PLT) states. Advanced liver disease states (DC, HCC, LT and PLT) were associated with HCV‐related mortality. The model was further stratified by HCV infection status, treatment uptake and SVR outcome (Figure 1b). HCV treatment was offered to patients in health states F0 to F4 as in the INTRO‐HCV trial, which resulted in either SVR with a possibility of reinfection, or therapeutic failure, whereby patients remain infected and may be retreated. Following treatment success, liver disease progression was assumed to halt during mild and moderate fibrosis (F0–F3) stages, and to reduce for more severe hepatic complications (F4/CC, DC, HCC). Age‐dependent mortality was also included.

### Model parameterization

Parameters relating to HCV treatment were obtained from the INTRO‐HCV trial [7] (Table 1). In the first year, the proportion of diagnosed individuals that were treated assumed the first‐year trial outcomes (integrated, 93.9%; standard‐of‐care, 72.0%). Following the first year, the treatment rates for the model were derived to give the total treated proportion in each arm of the trial (integrated, 98.0%; standard‐of‐care, 77.3%), yielding fitted second‐year treatment rates of 66.7% for the integrated pathway and 19.1% for the standard‐of‐care pathway (Figure S1), with no treatment occurring thereafter. Individuals, who have failed treatment, have been reinfected, or did not initiate HCV treatment would remain in the infected compartment and would be eligible for second‐year treatment. SVR rates (integrated, 94.6%; standard‐of‐care, 87.5%) also came from the trial. Uncertainty was associated with all trial‐derived parameters (Table 1). A low level of reinfection was assumed following successful treatment based on a recent study among PWID in Norway (3.7% [95% CI = 1.6%–7.2%] of SVR reinfected annually) [12]. Further details are in the Supporting Information.

**TABLE 1:** Base case parameters and annual transition probabilities used in the model.

	Baseline or fitted value/range, distribution for PSA	Source/comment
Demographic and epidemic parameters		
Background mortality of general population in Norway	Data from Norway life tables	Time‐varying age‐dependent mortality rate, adjusted to reflect the male (76.8%) and female (23.2%) ratio in the INTRO‐HCV trial. Probability of death by age was derived from Statistics Norway [8] life tables for 2017. Assume initial average age of cohort is 43 years.
Relative risk of death if currently injecting	17.52 (95% CI = 14.62–20.43), Normal	Adjust general population mortality in Norway for PWID during injecting duration by the SMR for PWID in Western Europe [9]
Average injecting duration	14 years (range, 4–24 years), Uniform	Point estimate from a Norwegian study, with +/−10 years assumed to account for uncertainty [10, 11]
HCV reinfection incidence rate among PWID	3.77 (95% CI = 1.63–7.42) per 100 person‐years, Lognormal Corresponding to 3.7% (95% CI = 1.6%–7.2%) reinfected per year	Reinfection estimated from a Norwegian study among individuals attending a low‐threshold clinic in Oslo who reported recent IDU in the past 3 months [12]. Reinfection incidence rate was converted to annual transition probabilities.
Treatment parameters		
Treated proportion in first year (F0–F4 only)	Fitted distribution: Integrated: 0.939 ( α=139,β=9), Beta Standard: 0.720 ( α=108,β=42), Beta	We fitted a Beta distribution with shape parameters derived from the proportion treated in the first year (integrated, 93.9%; standard, 72.0%) and the number intended to treat for each treatment pathway from the INTRO‐HCV trial [7].
Treatment in second year	Fitted distribution: Integrated: 0.667 ( α=6,β=3), Beta Standard: 0.191 ( α=8,β=34), Beta	In the second year, a background treatment rate was estimated by fitting a Beta distribution with shape parameters derived from the proportion treated following the first year, as determined by the total proportion treated in the INTRO‐HCV trial (integrated, 98.0%; standard, 77.3%). See Supporting Information for further details.
Proportion of individuals achieving SVR following DAA treatment	Integrated: 0.946 ( α=123,β=7), Beta Standard: 0.875 ( α=84,β=12), Beta	We fitted a Beta distribution with shape parameters derived from the proportion achieving SVR12 (integrated, 94.6%; standard, 87.5%) and the number initiating treatment and receiving a post‐treatment PCR test.

*Note*: Transitions to all‐cause mortality are also included.

Abbreviations: DAA, direct‐acting antivirals; F0–F4, METAVIR score for liver fibrosis stages; IDU, injecting drug use; INTRO‐HCV, integrated treatment of hepatitis C virus; METAVIR, meta‐analysis of histological data in viral hepatitis; PCR, polymerase chain reaction; PSA, probabilistic sensitivity analysis; PWID, people who inject drugs; SMR, standardized mortality ratio; SVR, sustained virological response.

Health state transition probabilities came from the literature [13, 14, 15] (Table S1) and adjusted for the INTRO‐HCV cohort’s higher proportion of HCV genotype 3 (~60%), which is associated with faster disease progression [16] (Supporting Information). Individuals with chronic HCV infection were assumed to progress through the disease stages [17].

Background death probabilities were derived from Norwegian life tables for 2017 [8], assuming the trial cohort’s average age at the start of the modelled cohort (43 years) and adjusting the mortality rate by the standardized mortality ratio for PWID in Western Europe [9] (17.5; 95% CI = 14.6–20.4) if currently engaged in IDU, assuming an average injection duration of 14 years [10, 11] (+/−10 years). Background death probabilities were adjusted for sex to reflect the male–female ratio in the INTRO‐HCV trial and changed as the cohort aged.

### Estimation of costs

All costs associated with HCV treatment were collected directly from the INTRO‐HCV trial using a healthcare perspective (Table 2, Table S2). An ingredients‐based micro‐costing approach was used where utilization of health services was identified in digital patient records (number of consultations with physician, psychologist, nurse or social worker; number of hospital in‐patient visits; number of lab tests; number of drugs administered; and number of integrated care meetings). Indirect capital, overhead costs and training costs were collected from financial records. One‐off treatment costs were comprised of fixed costs (training, elastography, ultrasound and building/infrastructure), DAA medication costs and other non‐DAA variable costs (human resources, consultation, pharmacy delivery and laboratory testing costs) and were calculated per patient treated for each treatment pathway. We assumed DAA list prices at baseline, with likely DAA price reductions being tested in sensitivity analyses to increase relevance. The annual unit costs of managing HCV‐related disease were estimated for this study using hospital records from an outpatient clinic (the OAT clinic LAR Laksevåg, Table 2). To account for uncertainty, total HCV‐related treatment costs were allowed to vary by +/−20%. Costs are presented in 2021 Euros (€), using 2021 purchasing power parities (PPP) for Norway with reference to the EU27 for health (€1 = 20.3 NOK) [8]. See further details in the Supporting Information.

**TABLE 2:** Health utilities and costs.

	Integrated	Standard	Source/comment
Treatment costs per patient			
DAA drug costs (12 weeks)	25 441.33	25 619.98	Variable cost of DAA medications only
Non‐DAA variable costs			Variable costs other than DAA drugs, broken down by costs of HR, consultations, pharmacy delivery and laboratory tests.
HR costs, consultations	79.79	46.83	
Pharmacy delivery cost	0.56	3.47	
Laboratory tests	59.04	59.04	
Fixed costs			Fixed costs, broken down by costs of training, elastography/ultrasound and building/infrastructure.
Training costs	37.26	17.72	
Elastography/ultrasound	184.22	342.24	
Building/infrastructure	NA	67.08	
Total treatment unit costs^a^ (per patient)	25 802.20	26 156.36	Sum of DAA drug costs, non‐DAA variable costs and fixed costs.
Annual healthcare costs^a^ for managing HCV‐related disease			
F0–F1	193		Annual unit costs for management of different HCV disease stages were estimated using hospital records from outpatient clinic LAR Laksevåg.
F2–F3	193		
F4	511		
DC	14 372		
HCC	20 093		
LT (transplantation)	65 987		One‐time cost of liver transplantation
LT	7946		Hospital/healthcare management costs incurred in year of liver transplantation
PLT	7946		
Health utilities			
Current injecting status			
Not currently injecting drugs	0.85 (uniform; 0.80–0.90)		Assumption based on [18] following injecting cessation.
Currently injecting drugs	0.73 (uniform; 0.68–0.78)		Reported index values for current PWID without chronic HCV infection [18, 19].
HCV‐related disease status			
F0–F1	Infected: 0.75 (IQR = 0.57–0.90); SVR: 0.79 (IQR = 0.59–0.92), triangular		Utility index values for mild fibrosis (F0–F1), moderate fibrosis (F2–F3) and compensated cirrhosis (F4) are derived from the INTRO‐HCV trial data [7].
F2–F3	Infected: 0.71 (IQR = 0.59–0.84); SVR: 0.78 (IQR = 0.69–0.88), triangular		
F4	Infected: 0.63 (IQR = 0.46–0.73); SVR: 0.73 (IQR = 0.50–0.84), triangular		
DC	0.45 ( α=123.75,β=151.25), Beta		Utility index values for advanced liver disease stages are derived from UK‐based studies [15, 20].
HCC	0.45 ( α=123.75,β=151.25), Beta		
LT	0.45 ( α=123.75,β=151.25), Beta		
PLT	0.67 ( α=59.2548,β=29.1852), Beta		
Discount rate	4% per annum		2012 Report from Norwegian Ministry of Finance [21]

*Note*: All costs are in 2021 € using PPP.

Abbreviations: DAA, direct‐acting antivirals; DC, decompensated cirrhosis; €, Euros; HCC, hepatocellular carcinoma; HCV, hepatitis C virus; HR, human resources; INTRO‐HCV, integrated treatment of hepatitis C virus; IQR, interquartile range; LT, liver transplant; NA, not applicable; PLT, post‐liver transplant; PPP, purchasing power parities; PWID, people who inject drugs; SVR, sustained virological response; UK, United Kingdom.

^a^ Total treatment unit cost per patient and all annual healthcare management costs have a +/−20% uncertainty associated with them and are each sampled from their respective uniform distributions.

### Derivation of health utilities

Health‐related quality of life health (HRQoL) measures were also collected from the INTRO‐HCV trial [7] using the EuroQoL EQ‐5D‐5L questionnaire [22] among patients with chronic HCV infection before treatment initiation and at 1 year following treatment success. These were stratified by fibrosis stage and converted into health utilities using a United Kingdom (UK) value set in the absence of a Norwegian value set (Table 2). Further details in Supporting Information. Remaining health utilities for post‐cirrhotic disease and current IDU status were derived from existing literature values (Table 2). Health utilities were sampled from their uncertainty distributions.

### Impact and cost‐effectiveness analysis

Starting with the initial distribution of patients by fibrosis stage as in the INTRO‐HCV trial, we simulated the model over the lifetime of the cohort (assuming a 50‐year time horizon) and compared the costs and health outcomes assuming all patients were engaged in either: (i) integrated treatment; (ii) standard‐of‐care treatment; or (iii) no treatment (counterfactual). The model tracked the number of patients in each HCV disease progression stage and infection, treatment or SVR state over time.

The total costs for each pathway consisted of the cumulative sum of testing, treatment, other intervention‐related costs and healthcare management costs over the time horizon. This was estimated by multiplying one‐off treatment unit costs (differs by integrated or standard‐of‐care pathway) to all HCV treatments given and annual unit costs of healthcare management to all HCV‐related disease states over the time horizon or until patient death.

The impact of treatment for each pathway was estimated in terms of total quality‐adjusted life‐years (QALYs) gained by applying the health utilities to their respective HCV disease progression states, with possible differences in health utilities depending on current injecting status (i.e. currently injecting or ceased injecting) and accumulating them annually over the time horizon, as described in previous studies [23, 24, 25]. Secondary outcomes were the differences in treatment uptake and proportion of incident liver disease complications prevented (F4/CC, DC and HCC and liver‐related deaths) between the integrated versus standard‐of‐care treatment pathways. Further details in Supporting Information.

The incremental cost‐effectiveness ratio (ICER) was estimated as the ratio of the difference in total costs between the integrated and standard‐of‐care treatment pathways divided by the corresponding difference in QALYs [26], which represented the incremental costs of integrated treatment associated per QALY gained from the health provider’s perspective over a lifetime time horizon. All costs and QALYs were discounted at 4% per annum [21, 27]. The analysis did not follow a preregistered analysis plan, but used similar methods to our previous studies [24, 28, 29]. This study has been reported as per the Consolidated Health Economic Evaluation Reporting Standards (CHEERS) guidelines (Checklist S1).

### Sensitivity analyses

To account for uncertainty in model parameters, we conducted a probabilistic sensitivity analysis by simultaneously sampling all model parameters 1000 times from their respective statistical distributions (Table 1) and simulating the model for each sampled parameter set. Because DAA medication costs as provided by the Norwegian Health Services are confidential, we evaluated the ICER for different reductions in the DAA list price (30%/60%/90%). To determine the probability of the integrated pathway being cost‐effective compared to the standard‐of‐care pathway, the estimated ICERs were assessed against a conventional (€70 000/QALY) willingness‐to‐pay (WTP) threshold suggested by the Norwegian Directorate of Health [30] and a lower (€20 000/QALY) threshold commonly considered by other high‐income countries [31]. The probability of integrated treatment being cost‐saving was also assessed.

We performed further univariate sensitivity analyses to investigate how the ICER changed by varying key parameters in the model. These included assumptions on total treatment unit cost (half or double the baseline cost), reinfection incidence (2/100 person‐years or 12.1/100 person‐years), second‐year treatment rate (integrated pathway same as standard‐of‐care), treatment duration (none or continued treatment for 10 years), average age of cohort (20 years younger or 10 years older), initial distribution of disease states (less or more severe disease states compared to baseline), injecting duration (5 or 30 years), healthcare management costs (none or double healthcare costs), whether SVR differed by pathway (no difference compared to higher SVR rate for integrated pathway at baseline), health utilities (UK‐based literature values compared to trial values) [14, 15], time horizon (25 years compared to 50 years at baseline), discounting (none or double the baseline rate) and currency conversion (lower or higher rates or using market exchange rates). Further details are in Table S3.

We then performed additional sensitivity analyses to investigate the cost‐effectiveness of the integrated treatment pathway under different assumptions on the costs of service delivery and care, using cost data from other country settings. Specifically, we considered settings with (scenario S1) similar treatment costs, but lower healthcare management costs for late‐stage HCV‐related disease (using costs from United Kingdom); (scenario S2) higher treatment costs (using costs from United States [US]); (scenario S3) lower treatment costs, but higher healthcare costs (using costs from France); and (scenario S4) lower costs of both treatment and healthcare management (using costs from Australia). For these sensitivity analyses, we assumed the same trial effects for the integrated and standard‐of‐care treatment pathways, but used the corresponding cost assumptions from published cost‐effectiveness studies for other settings (Table 3, Table S4). Actual negotiated DAA prices are confidential, but are likely to be lower than the list price so, for these analyses, we considered DAA medications at list price and also 30%/60%/90% price reductions. Meanwhile, annual healthcare costs for managing HCV‐related disease were adjusted by producer price indices (PPI) to the year 2021. The median ICER and probability of being cost‐effective or cost‐saving for integrated treatment were evaluated for each country example, with all costs converted to 2021 Euros (€) using PPP for cross‐country comparison.

**TABLE 3:** Mean costs used in sensitivity analyses estimating the cost‐effectiveness of integrated versus standard‐of‐care treatment in settings with different conditions, using selected countries as case examples.

	Scenario S0 (Norway)	Scenario S1 (UK)	Scenario S2 (US)	Scenario S3 (France)	Scenario S4 (Australia)	Source/comment
Treatment costs per patient						
DAA drug costs (12 week)						
DAA list price	25 441	38 171	56 699	28 730	6043	DAA list prices obtained from selected country studies representing each scenario: UK [24], US [32], France [33], Australia [34, 35].
30% Reduction	17 809	26 720	39 689	20 111	4230	
60% Reduction	10 177	15 268	22 680	11 492	2417	
90% Reduction	2544	3817	5670	2873	604	
Other HCV‐related treatment costs^a^						
	361	407	2122	790	906	Other HCV‐related treatment costs besides DAA drugs, including non‐DAA variable costs and fixed costs (refer to Table 1), adjusted to 2021 using PPI.
Total treatment unit costs (per patient)						
DAA list price	25 802	38 579	58 821	29 520	6949	Sum of DAA drug costs and other treatment costs.
30% Reduction	18 170	27 127	41 811	20 901	5136	
60% Reduction	10 537	15 676	24 801	12 282	3323	
90% Reduction	2905	4224	7792	3663	1511	
Annual healthcare costs^a^ for managing HCV‐related disease						
F0–F1	193	208	606	810	224	Published cost‐effectiveness studies: UK [14, 36], US [37], France [33, 38], Australia [34].
F2–F3	193	1083	613	910	224	
F4	511	1719	1452	8828	469	
DC	14 372	13 774	16 138	28 631	7622	
HCC	20 093	2275	29 678	31 259	5395	
LT (transplantation)	65 987	41 278	85 817	161 135	72 983	One‐time cost of liver transplantation
LT	7946	14 285	^b^	^b^	^b^	Hospital/healthcare management costs incurred in year of liver transplantation
PLT	7946	2092	22 521	23 542	^b^	

*Note*: Costs for HCV treatment and annual healthcare management for HCV‐related disease were obtained from published estimates for each setting and converted to 2021 € using PPP for the analyses. Reductions in DAA medication costs are in relation to the published list prices. Unadjusted point estimates and uncertainty distributions for the various cost components for each country in local currency are described in Table S4.

Abbreviations: DAA, direct‐acting antivirals; DC, decompensated cirrhosis; €, Euros; HCC, hepatocellular carcinoma; HCV, hepatitis C virus; LT, liver transplant; PLT, post‐liver transplant; PPI, producer price index; PPP, purchasing power parities; UK, United Kingdom; US, United States.

^a^ Non‐DAA‐related HCV treatment costs and annual healthcare costs for chronic HCV disease stages in each country were obtained from published country‐level studies and adjusted for inflation using PPI to 2021 from cost year in which the original study occurred, if known. Further details are in Table S4.

^b^ Assumed to be combined with one‐time LT costs because annual costs were not available from published studies.

Model results have been presented as the median and 95% uncertainty interval (UI) of 1000 model simulations. All model analyses were performed in MATLAB (version 2021a).

## RESULTS

### Costing analysis

The total unit costs per patient treated for the integrated treatment pathway was €25 802/patient (Table 2) and comprised primarily (98.6%) of DAA medication costs (€25 441/patient), other non‐DAA variable costs (€139/patient) and fixed costs (€221/patient). This was slightly lower compared to the standard‐of‐care pathway (€26 156/patient), which had higher fixed costs (€427/patient). Annual healthcare management costs varied by HCV disease progression stage from €193/year for pre‐cirrhotic (F0–F3) disease stages to €20 093/year for HCC, with liver transplantation requiring a one‐time cost of €65 987 and €7946/year for managing liver transplant patients (Table 2).

### HRQoL analysis

Analysis of the HRQoL data from the INTRO‐HCV trial revealed lower median utilities for increasing fibrosis scores at treatment initiation, namely, F0–F1: 0.75 (interquartile range [IQR] = 0.57–0.90), F2–F3: 0.71 (IQR = 0.59–0.84), F4: 0.63 (IQR = 0.46–0.73). These improved at successful post‐treatment follow‐up, F0–F1: 0.79 (IQR = 0.59–0.92), F2–F3: 0.78 (IQR = 0.69–0.88), F4: 0.73 (IQR = 0.50–0.84) (Table 2).

### Cost of HCV treatment pathways

The standard‐of‐care pathway was estimated to cost €31 093 (95% UI = 24 314–38 756) per person diagnosed and eligible for HCV treatment over 50‐years, with two thirds of the costs due to HCV treatment (68.4%) and the remainder due to healthcare management (31.6%) (Table S5). The integrated pathway was estimated to cost €34 043 (95% UI = 27 123–41 601) per diagnosed person eligible for HCV treatment (Table 4), with a slightly greater proportion of the costs due to HCV treatment (76.0%) than healthcare management (24.0%) over the 50‐year time horizon. These total costs decrease by a quarter (23%) or half (45%) if the DAA drugs are 30% or 60% cheaper, with the proportion of total costs due to HCV treatment decreasing to 69.2% or 56.6%, respectively (Table S5). Compared to the standard‐of‐care pathway, the integrated pathway increased costs by €2884 (95% UI = 928–5562) per diagnosed person at DAA list price, with this reducing to €1569 (95% UI = −9 to 3106) or €210 (95% UI = −1,182 to 1256) per diagnosed person for a 30% or 60% reduction in DAA drug costs, respectively.

**TABLE 4:** ICERs for the integrated treatment pathway compared to the standard treatment pathway, at baseline and for specific reductions in DAA price (30%/60%/90%).

	Costs per person diagnosed (€)		QALYs per person diagnosed		ICER	Probability	Probability
	Total	Incremental	Total	Incremental	Cost/QALY gained	Cost‐effective	Cost‐saving
DAA list price (€25 400 for 12 weeks)							
Standard	31 093 (24 314–38 756)	–	9.6 (7.8–12.2)	–	–	–	–
Integrated	34 043 (27 123–41 601)	2884 (928–5562)	9.8 (7.9–12.6)	0.2 (0.1–0.5)	13 272	98.6% (conventional) 71.4% (lower)	0.3%
30% DAA price reduction (€17 800 for 12 weeks)							
Standard	24 548 (20 621–30 671)	–	9.6 (7.8–12.2)	–	–	–	–
Integrated	26 262 (22 912–31 378)	1569 (−9 to 3106)	9.8 (7.9–12.6)	0.2 (0.1 to 0.5)	6910	99.2% (conventional) 91.4% (lower)	2.6%
60% DAA price reduction (€10 200 for 12 weeks)							
Standard	18 398 (14 437–24 363)	–	9.6 (7.8 to 12.2)	–	–	–	–
Integrated	18 618 (15 323–23 756)	210 (−1182 to 1256)	9.8 (7.9–12.6)	0.2 (0.1–0.5)	929	99.6% (conventional) 99.1% (lower)	36.1%
90% DAA price reduction (€2500 for 12 weeks)							
Standard	12 226 (8093–18 011)	–	9.6 (7.8 –12.2)	–	–	–	–
Integrated	10 962 (7668–16 099)	−1134 (−2610 to −330)	9.8 (7.9–12.6)	0.2 (0.1–0.5)	Cost‐saving	99.9% (conventional) 99.6% (lower)	99.6%

*Note*: Costs and QALYs are discounted at a rate of 4.0% per annum. Time horizon is 50 years. Total and incremental costs and QALYs are divided by the total number of individuals (*n* = 298) to report outcomes per person that was diagnosed, and are shown as the median and 95% UI of 1000 model simulations. The probability of being cost‐effective is the proportion of model simulations that are below the conventional (€70 000 per QALY gained) and lower (€20 000 per QALY gained) willingness‐to‐pay thresholds. Costs are presented in 2021 €.

Abbreviations: DAA, direct‐acting antivirals; €, Euros; ICERs, incremental cost‐effectiveness ratios; QALYS, quality‐adjusted life‐years; UI, uncertainty interval.

### Health impact of treatment

Overall, the integrated pathway was associated with an additional 0.2 (95% UI = 0.1–0.5) QALYs gained per diagnosed person compared to the standard‐of‐care pathway over the 50‐year time horizon (Table 4). The integrated pathway resulted in a 23.0% (95% UI = 11.7%–37.4%) and 32.0% (95% UI = 19.3%–51.3%) increase in the number of PWID initiating treatment and achieving SVR, respectively, and averted 12% to 13% of new end‐stage liver disease (ESLD) cases and HCV‐related deaths over 50 years (Figures S2‐S3, Tables S6‐S7).

### Cost‐effectiveness of integrated treatment pathway

**FIGURE 2:**
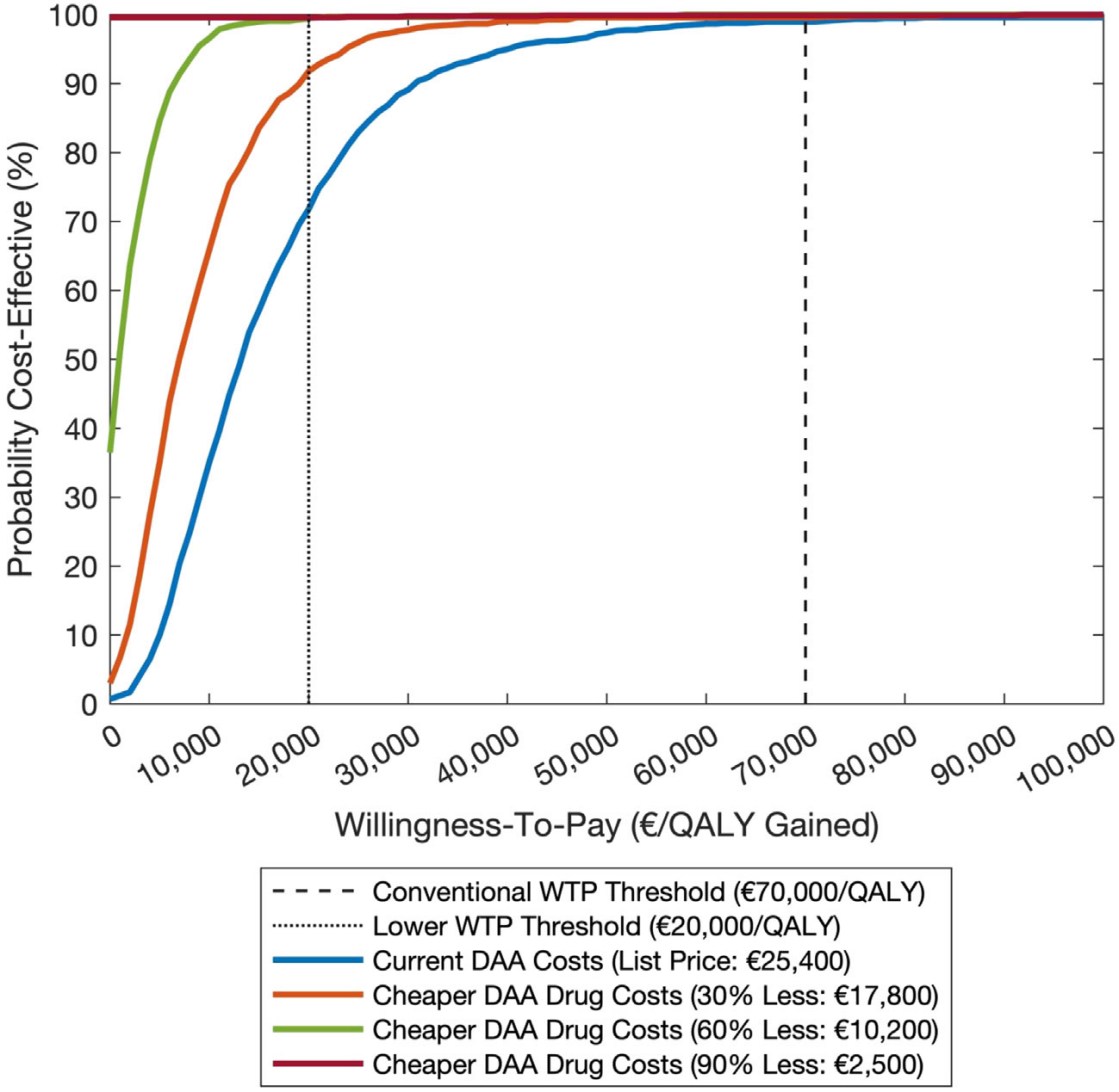
Cost‐effectiveness acceptability curve (CEAC) showing the probability of the integrated treatment pathway being cost‐effective compared to standard‐of‐care pathway for two willingness‐to‐pay (WTP) thresholds. The plots are shown for the baseline scenario as well as for selected reductions in direct‐acting antivirals (DAA) medication prices of 30%/60%/90%. Costs and quality‐adjusted life‐years (QALYs) are discounted at a rate of 4.0% per annum. Time horizon is 50 years. The conventional (dashed line) and lower (dotted line) WTP thresholds are shown. Results are for 1000 model simulations.

Compared to the standard‐of‐care treatment pathway, the integrated treatment pathway resulted in an ICER of €13 272 per QALY gained (Table 4, Table S8), with a 98.6% and 71.4% probability of being cost‐effective against the €70 000/QALY and €20 000/QALY WTP threshold, respectively, and a 0.3% probability of being cost‐saving (Figure 2).

**FIGURE 3:**
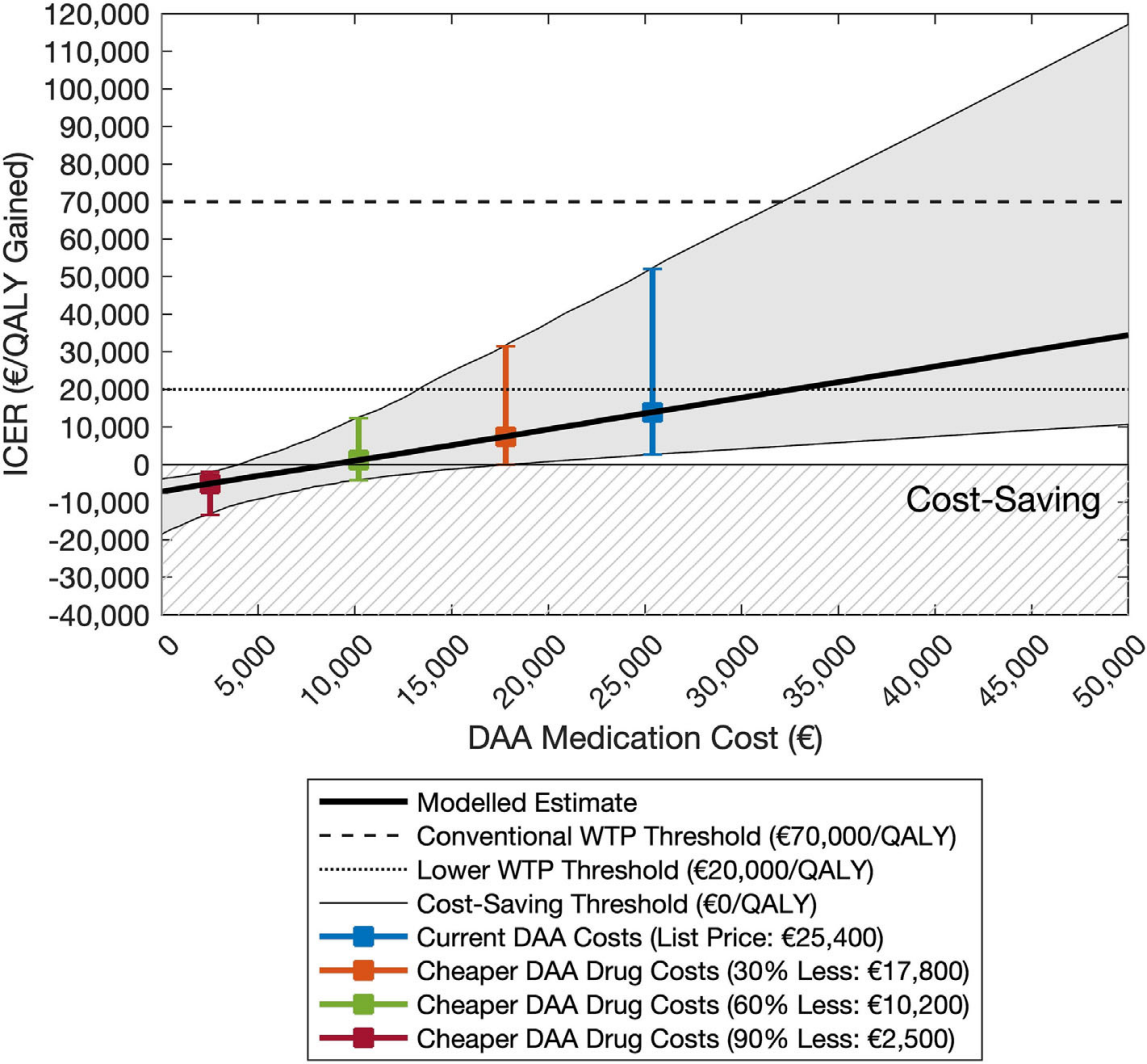
Probabilistic sensitivity analyses showing the effects of varying the price of direct‐acting antivirals (DAA) medications on the incremental cost‐effectiveness ratio (ICER) of the integrated treatment pathway compared to standard treatment pathway. Costs and quality‐adjusted life‐years (QALYs) are discounted at a rate of 4.0% per annum. Time horizon is 50 years. Results are for 1000 model simulations. The shaded regions indicate the 95% uncertainty interval (UI) for the ICER across varying DAA medication costs. Areas with hatching indicate cost‐savings and a positive health impact, and so where integrated treatment is dominant.

The price of DAA medications had a strong effect on the ICER (Figure 3). For a 30% reduction in DAA medication price (€17 800 for 12 weeks), the ICER was halved (€6910 per QALY gained) and the probability of integrated treatment being cost‐effective at the lower WTP threshold increased to 91.4%. With a 60% reduction in DAA medication cost (€10 200 for 12 weeks) there was a >99% probability of being cost‐effective at both WTP thresholds, and a 36.1% probability of being cost‐saving. This increased to a 99.6% probability of being cost‐saving with a 90% reduction in DAA medication cost (€2500 for 12 weeks). The cost‐effectiveness plane, cost‐effectiveness probabilities and ICER for different DAA drug prices are shown in Figures S4‐S6.

### Univariate sensitivity analyses

**FIGURE 4:**
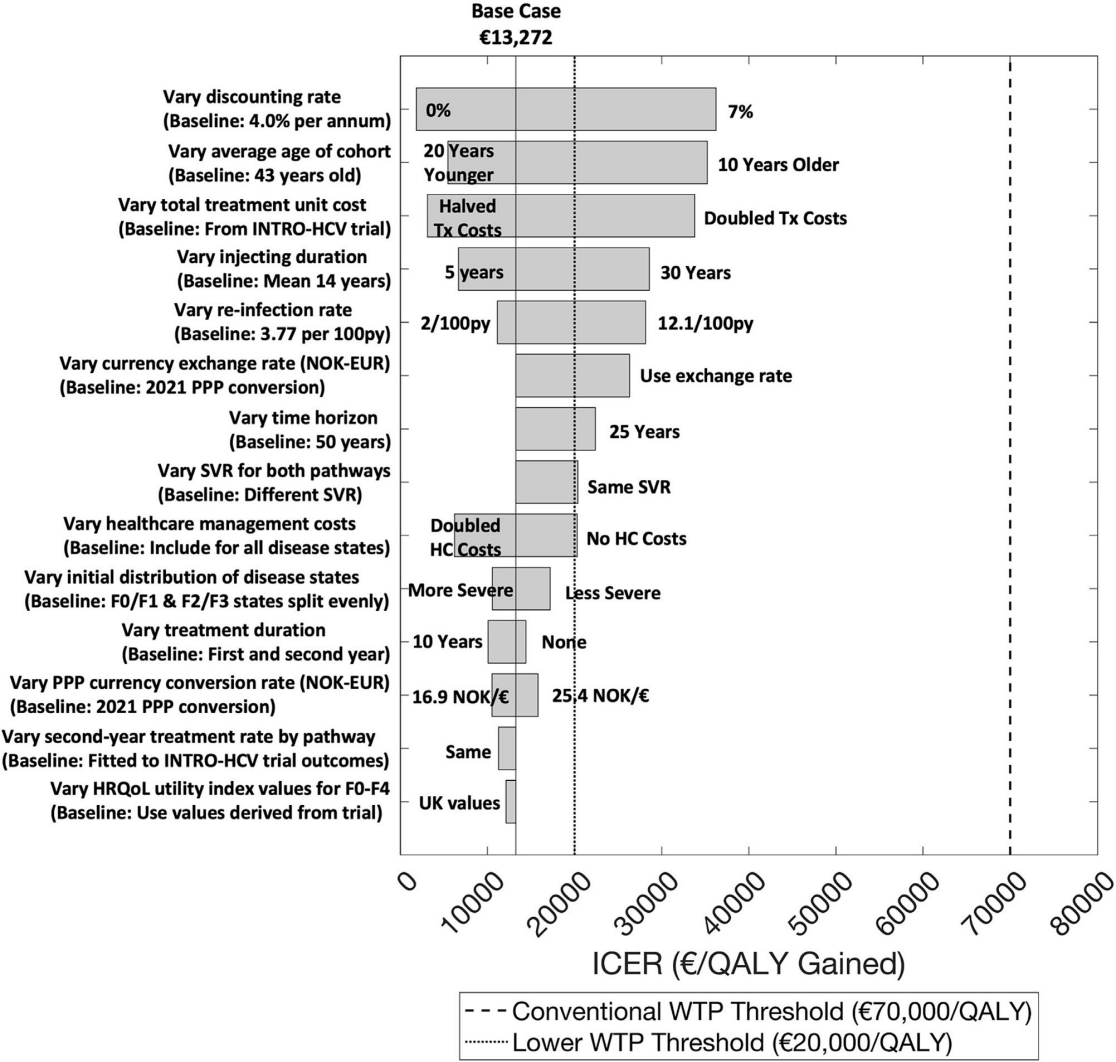
Univariate sensitivity analyses on the incremental cost‐effectiveness ratio (ICER) of the integrated treatment pathway compared to the standard‐of‐care treatment pathway, assuming list costs for direct‐acting antivirals (DAA) treatments (€47 800 per 12‐week treatment). Costs and quality‐adjusted life‐years (QALYs) are discounted at a rate of 4.0% per annum. Time horizon is 50 years. The conventional (dashed line) and lower (dotted line) willingness‐to‐pay (WTP) thresholds are shown. Results are for 1000 model simulations.

With DAAs at list price, univariate sensitivity analyses suggested that the ICER for the integrated treatment pathway was strongly affected by assumptions on the discount rate, average cohort age (especially older age), treatment unit costs, injecting duration and reinfection rate. However, for all these scenarios, the ICER remained below the conventional €70 000/QALY WTP threshold, with the probability of integrated treatment being cost‐effective ranging between 80% and 100% (Figure 4). Smaller changes occurred for other parameter assumptions. Similar results were found assuming a 60% reduction in DAA price (Figure S7).

### Variation in cost‐effectiveness of integrated treatment across selected settings

**FIGURE 5:**
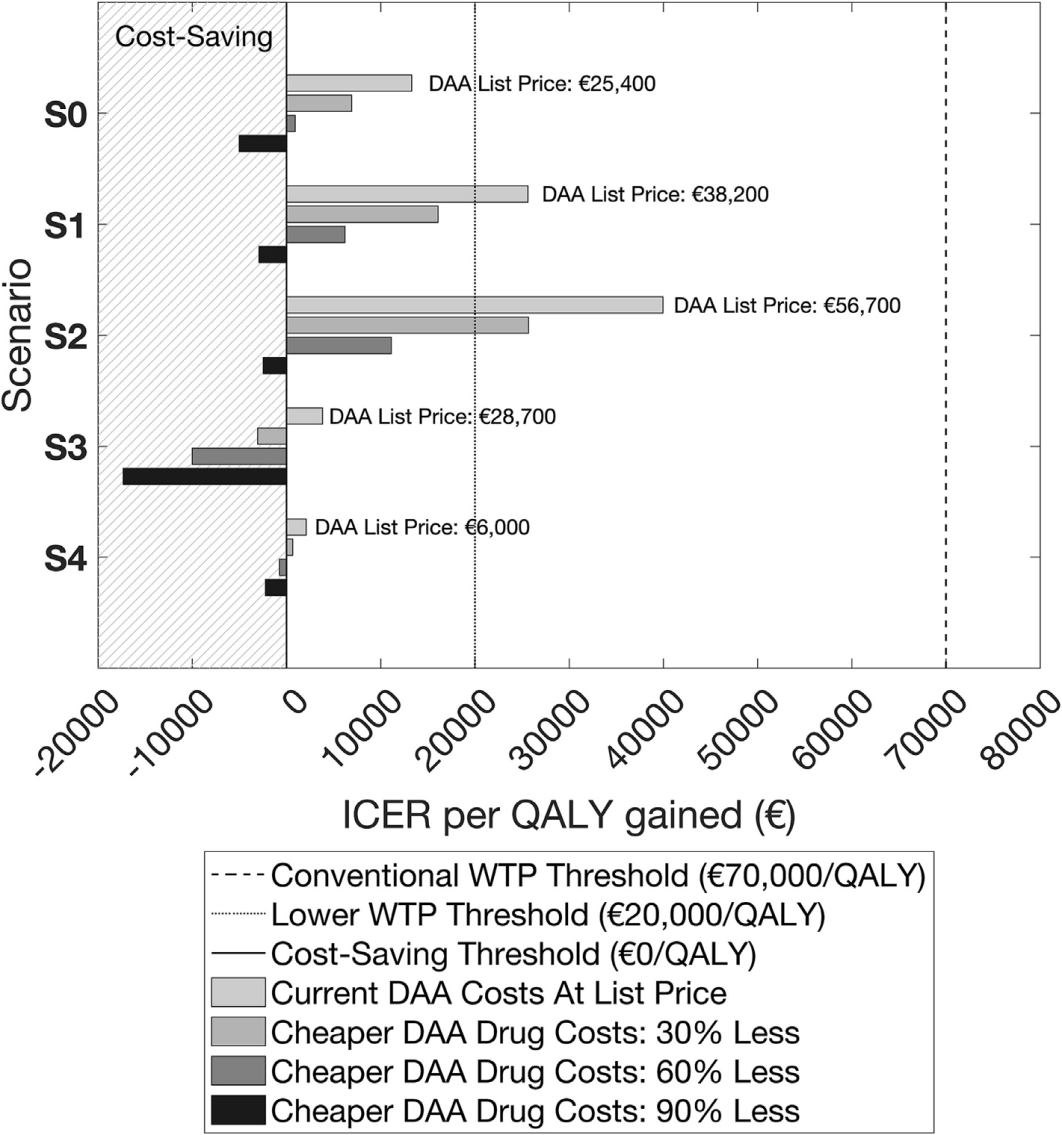
Modelled estimates for the incremental cost‐effectiveness ratio (ICER) of delivering integrated hepatitis C virus (HCV) treatment compared to standard‐of‐care treatment as in the integrated treatment of (INTRO)‐HCV trial for selected countries. Various pricing scenarios on direct‐acting antivirals (DAA) medication costs are considered (namely, DAA costs at current list price and reductions of 30%, 60% and 90%), whereas non‐DAA‐related HCV treatment costs and healthcare management costs are adjusted to 2021 for inflation by producer price index (PPI), with total treatment unit costs presented in 2021 Euros (€) for comparison across scenarios. Costs and quality‐adjusted life‐years (QALYs) are discounted at a rate of 4.0% per annum. Time horizon is 50 years. Results are for 1000 model simulations. Areas with hatching indicate cost‐savings and a positive health impact, and so where integrated treatment is dominant.

Across the selected settings, at DAA list prices the ICER for integrated treatment compared to standard‐of‐care treatment (Norway [scenario S0]: €13 272/QALY gained) ranged from €39 966/QALY gained (scenario S2) and €25 623/QALY gained (scenario S1) down to €3819/QALY gained (scenario S3) and €2089/QALY gained (scenario S4) (Figure 5). In all settings, integrated treatment was cost‐effective with high probability (>80%) at the €70 000/QALY WTP threshold (Figure S8). With a 30% reduction in DAA price, all settings except scenario S2 were cost‐effective (>60% probability) at the €20 000/QALY WTP threshold, whereas a 60% DAA price reduction made all settings cost‐effective at this lower WTP threshold. A 90% DAA price reduction resulted in integrated treatment being cost‐saving with high probability (>95%) for all settings except scenario S2 (79% probability).

## DISCUSSION

In this study, we used a health economic modelling framework to estimate the long‐term health outcomes and costs of implementing integrated versus standard HCV treatment delivery as undertaken in the INTRO‐HCV intervention trial conducted in Norway. We estimated that through increasing the number of PWID accessing treatment and achieving SVR, integrated treatment would avert 12% to 13% additional cases of advanced liver disease and HCV‐related deaths over a 50‐year time horizon. At existing list prices for DAAs, the integrated pathway was estimated to cost €13 272 per QALY gained compared to the standard‐of‐care pathway, making it highly cost‐effective (>98% probability) at the €70 000/QALY WTP threshold for Norway. Moderate reductions in DAA medication price (e.g. 30% or 60% cheaper than list price), as are likely to have been achieved through price negotiations, substantially improved the cost‐effectiveness of integrated treatment, including at lower WTP thresholds considered by other high‐income countries (€20 000/QALY), potentially making the intervention cost‐saving.

Implementing effective and cost‐effective strategies to optimize the HCV treatment cascade‐of‐care is critical to improving HCV‐related disease outcomes among PWID and reducing the incidence of infection. In Norway, the integrated pathway delivered HCV treatment on‐site in community OAT clinics and care centers managed by a multidisciplinary healthcare team, which led to improvements in HCV treatment initiation, retention and SVR outcome compared to the standard‐of‐care pathway of referral to outpatient clinics for HCV treatment. These findings have led to integrated HCV treatment recently being adopted into routine healthcare practice among PWID in community settings in Norway [39]. Our results are potentially generalizable to other settings with similar OAT provision infrastructures for PWID and suggest ways that an integrated approach to delivering HCV treatment can be implemented in these settings.

In sensitivity analyses, we found that integrated treatment was still likely to be cost‐effective in all settings considered if there were moderate decreases in HCV treatment costs, which price negotiations are likely to have achieved. This includes settings with lower or higher treatment costs (e.g. scenarios S2 and S4), or lower and higher healthcare costs (e.g. scenarios S1 and S3). This emphasizes the generalizability of our study results in settings with particular characteristics, suggesting the potential benefits of expanding integrated HCV treatment in other settings.

### Strengths and limitations

The main strength of our study is its extensive use of detailed primary data on the impact, health benefits and costs of implementing integrated HCV treatment among PWID in Norway; primarily linked to the INTRO‐HCV randomized controlled trial. This included the detailed estimation of treatment costs in OAT clinics that captures all operational costs of providing HCV treatment, and the estimation of costs for managing HCV‐related disease from outpatient hospital records. Moreover, we derived health utilities for before and after HCV treatment, collected using EuroQoL EQ‐5D‐5L questionnaires, which showed that achieving SVR led to improvements in quality of life as found in other studies [40, 41].

Limitations include the negotiated costs of DAA medications being confidential in Norway and so we used list prices in our baseline model projections. DAA medication prices have dropped substantially globally over recent years and are very likely to be less than list prices in Norway, with our sensitivity analyses showing that cheaper DAA prices markedly improve the cost‐effectiveness of integrated treatment. Second, although the standard‐of‐care treatment pathway was primarily referral‐based in terms of HCV treatment, extended diagnostic elements were integrated into this pathway, which increased the cost compared to a more minimalistic model without integrated diagnostics. This reduced the differences in total costs between integrated and standard‐of‐care treatment pathways and possibly also reduced the differences in SVR. However, with the shift to universal healthcare occurring in Norway and across many high‐income countries, standard‐of‐care referral pathways will likely start including some level of integration into health services. Third, intervention costs were only estimated for Bergen and Stavanger; however, these costs are likely representative of Norway. Last, although our modelling framework included reinfection, it did not incorporate the prevention benefits of treatment, meaning our cost‐effectiveness estimates may be conservative.

### Comparison with other studies

Few studies have investigated the cost‐effectiveness of integrated approaches to HCV treatment for PWID in OAT settings. The Hepatitis C Awareness Through to Treatment (HepCATT) intervention in the United Kingdom found that using a nurse facilitator to increase testing and treatment referral among PWID attending drug treatment centers was cost‐effective compared to the standard‐of‐care with no nurse‐led facilitation [24]. However, in HepCATT, all HCV treatments were referred to hospital and so did not include community treatment, unlike our analyses where the integrated pathway included treatment provided in the community OAT clinics or CCCs. A study among PWID in the United States suggested that different modalities of on‐site HCV treatment in OAT programs (e.g. group treatment, directly observed therapy) were likely to be highly cost‐effective compared to off‐site testing and treatment referral [32]. Moreover, both the HepCATT and US studies started in the pre‐DAA era, and the use of non‐DAA medications could have adversely affected participation and worsened the cost‐effectiveness. A previous study conducted in Norway [30] investigated the cost‐effectiveness of DAA treatment compared with older interferon‐based drug regimens from a Norwegian healthcare perspective, however, it did not consider different modes of delivery.

## CONCLUSIONS AND IMPLICATIONS

With the advent of DAA medications, the means to eliminate HCV are within our grasp, yet there remain challenges to ensuring that these DAAs have the greatest impact, with PWID being a high priority group that has considerable barriers to accessing conventional healthcare. Integrating the delivery of HCV treatment for PWID in community settings is likely to be an effective and cost‐effective approach for increasing treatment uptake and completion within this marginalized group in many settings and may even be cost‐saving if drug prices are negotiated to <60% of list prices. Integrated care is now adopted into routine healthcare practice among PWID in community settings in Norway. Implementation of such integrated HCV care pathways in Norway and other settings with similar HCV treatment infrastructure could improve treatment uptake and health outcomes among HCV‐infected PWID.

## AUTHOR CONTRIBUTIONS

Aaron Guanliang Lim, Christer Frode Aas, Lars Thore Fadnes, Peter Vickerman and Kjell Arne Johansson designed the cost‐effectiveness study. Ege Su Çağlar collected and analyzed the cost data together with Christer Frode Aas and Kjell Arne Johansson. Aaron G. Lim and Christer Frode Aas analyzed the HRQoL data, with help from Jørn Henrik Vold. Aaron G. Lim developed the model and conducted all modelling analyses, with guidance from Lars Thore Fadnes, Peter Vickerman and Kjell Arne Johansson. Aaron G. Lim wrote the initial draft of the manuscript. All authors contributed to guiding the overall analysis plan, interpreting interim and final results and critically reviewing the final version of the manuscript.

## DECLARATION OF INTERESTS

P.V. has received unrestricted research grants from Gilead unrelated to this work. All other authors declare no competing interests.

## ETHICS STATEMENT

The INTRO‐HCV study including this modelling analysis was approved by the regional ethical committee in Norway (2017/51/REK Vest).

## Supporting information


**Figure S1.** Model input parameters for background treatment rate.
**Figure S2.** Selected modelled health states over time.
**Figure S3.** HCV cascade of care and HCV burden.
**Figure S4.** Cost‐effectiveness plane at baseline and selected reductions in DAA price.
**Figure S5.** Effect of DAA medication price on probability of cost‐effectiveness.
**Figure S6.** ICER by varying DAA medication cost.
**Figure S7.** Univariate sensitivity analyses with 60% DAA price reduction.
**Figure S8.** Probability of integrated treatment being cost‐effective or cost‐saving.
**Table S1.** Annual health state transition probabilities and initial conditions.
**Table S2.** HCV treatment costs and annual healthcare costs in 2021 NOK.
**Table S3.** Details of univariate sensitivity analyses undertaken.
**Table S4.** Parameter estimates and uncertainty for the various cost components in selected countries.
**Table S5.** Breakdown of total costs by treatment and healthcare management by DAA price reduction.
**Table S6.** Cascade‐of‐care at baseline.
**Table S7.** Total and incremental averted HCV‐related morbidity and mortality for each scenario.
**Table S8.** Incremental cost‐effectiveness ratios (ICERs) at baseline per diagnosed person.
**Checklist C1.** Consolidated Health Economic Evaluation Reporting Standards (CHEERS) 2022.

## Data Availability

The data that support the findings of this study are available from the corresponding author upon reasonable request. The data are not publicly available due to privacy or ethical restrictions.
