# Supplementary material for: Cost‐effectiveness of integrated treatment for hepatitis C virus (HCV) among people who inject drugs in Norway: An economic evaluation of the INTRO‐HCV trial
Source: Addiction. 2023 Jul 29;118(12):2424–39. doi: 10.1111/add.16305 (PMC10952903; doi:10.1111/add.16305)
Supplement: Supplementary file 1 — Figure S1. Model input parameters for background treatment rate. Figure S2. Selected modelled health states over time. Figure S3. HCV cascade of care and HCV burden. Figure S4. Cost‐effectiveness plane at baseline and selected reductions in DAA price. Figure S5. Effect of DAA medication price on probability of cost‐effectiveness. Figure S6. ICER by varying DAA medication cost. Figure S7. Univariate sensitivity analyses with 60% DAA price reduction. Figure S8. Probability of integrated treatment being cost‐effective or cost‐saving. Table S1. Annual health state transition probabilities and initial conditions. Table S2. HCV treatment costs and annual healthcare costs in 2021 NOK. Table S3. Details of univariate sensitivity analyses undertaken. Table S4. Parameter estimates and uncertainty for the various cost components in selected countries. Table S5. Breakdown of total costs by treatment and healthcare management by DAA price reduction. Table S6. Cascade‐of‐care at baseline. Table S7. Total and incremental averted HCV‐related morbidity and mortality for each scenario. Table S8. Incremental cost‐effectiveness ratios (ICERs) at baseline per diagnosed person. Checklist C1. Consolidated Health Economic Evaluation Reporting Standards (CHEERS) 2022. [file ADD-118-2424-s001.docx]

**Supplementary Materials**

**Cost-effectiveness of integrated treatment for hepatitis C virus (HCV) among people who inject drugs in Norway: An economic evaluation of the INTRO-HCV trial**

**Authors:**

Aaron G Lim^1^, DPhil

Christer Frode Aas^2,3,4^, PhD

Ege Su Çağlar^2,3^, MPhil

Jørn Henrik Vold^2,3,4^, PhD

Lars Thore Fadnes^2,3^*, PhD

Peter Vickerman^1,^*, DPhil

Kjell Arne Johansson^2,3,^*, PhD

*Joint senior authors

^1^Population Health Sciences, Bristol Medical School, University of Bristol, Bristol, UK

^2^Bergen Addiction Research, Department of Addiction Medicine, Haukeland University Hospital, Bergen, Norway

^3^Department of Global Public Health and Primary Care, University of Bergen, Bergen, Norway

^4^Division of Psychiatry, Haukeland University Hospital, Bergen, Norway

**Corresponding Author**: Dr Aaron G Lim, DPhil, Population Health Sciences, Bristol Medical School, University of Bristol, Oakfield House, Oakfield Grove, Clifton, BS8 2BN, UK. [aaron.lim@bristol.ac.uk](mailto:Aaron.lim@bristol.ac.uk). +44 (0) 117 455 4129

Table of Contents

[Supplementary Methods 3](#_Toc135384866)

[Summary of costing methodology 3](#_Toc135384867)

[Setting for OAT and transition to INTRO-HCV 3](#_Toc135384868)

[Cost assessments 3](#_Toc135384869)

[Cost inputs: Variables costs and fixed costs 3](#_Toc135384870)

[Initial distribution of participants by health state 5](#_Toc135384871)

[Determining treatment rates 5](#_Toc135384872)

[Adjustment of higher proportion of HCV genotype 3 6](#_Toc135384873)

[Non-adjustment of SMR for HCV-related death 6](#_Toc135384874)

[Utility index values from trial data 6](#_Toc135384875)

[Applying health utilities 7](#_Toc135384876)

[Univariate sensitivity analysis and comparison of different settings 7](#_Toc135384877)

[Supplementary Results 8](#_Toc135384878)

[Total costs and QALYs 8](#_Toc135384879)

[Treatment uptake and cascade-of-care 8](#_Toc135384880)

[Disease burden 8](#_Toc135384881)

[Reductions in DAA prices improve cost-effectiveness substantially 8](#_Toc135384882)

[Supplementary Figures 10](#_Toc135384883)

[Supplementary Figure S1. Model input parameters for background treatment rate. 10](#_Toc135384884)

[Supplementary Figure S2. Selected modelled health states over time. 10](#_Toc135384885)

[Supplementary Figure S3. HCV cascade of care and HCV burden. 11](#_Toc135384886)

[Supplementary Figure S4. Cost-effectiveness plane at baseline and selected reductions in DAA price. 12](#_Toc135384887)

[Supplementary Figure S5. Effect of DAA medication price on probability of cost-effectiveness. 13](#_Toc135384888)

[Supplementary Figure S6. ICER by varying DAA medication cost. 14](#_Toc135384889)

[Supplementary Figure S7. Univariate sensitivity analyses with 60% DAA price reduction. 15](#_Toc135384890)

[Supplementary Figure S8. Probability of integrated treatment being cost-effective or cost-saving. 18](#_Toc135384891)

[Supplementary Tables 19](#_Toc135384892)

[Supplementary Table S1. Annual health state transition probabilities and initial conditions. 19](#_Toc135384893)

[Supplementary Table S2. HCV treatment costs and annual healthcare costs in 2021 NOK. 20](#_Toc135384894)

[Supplementary Table S3. Details of univariate sensitivity analyses undertaken. 21](#_Toc135384895)

[Supplementary Table S4. Parameter estimates and uncertainty for the various cost components in selected countries. 22](#_Toc135384896)

[Supplementary Table S5. Breakdown of total costs by treatment and healthcare management by DAA price reduction. 24](#_Toc135384897)

[Supplementary Table S6. Cascade-of-care at baseline. 25](#_Toc135384898)

[Supplementary Table S7. Total and incremental averted HCV-related morbidity and mortality for each scenario. 26](#_Toc135384899)

[Supplementary Table S8. Incremental cost-effectiveness ratios (ICERs) at baseline per diagnosed person. 27](#_Toc135384900)

[Supplementary Checklist C1. Consolidated Health Economic Evaluation Reporting Standards (CHEERS) 2022. 28](#_Toc135384901)

[Supplementary References 31](#_Toc135384902)

Supplementary Methods

Summary of costing methodology

Setting for OAT and transition to INTRO-HCV

Data for patients on opioid agonist therapy (OAT) were collected from one of the seven OAT clinics in Bergen, Norway. Patients were identified through their responsible health personnel from registration and all patients that were enrolled in the OAT clinic during 2017 were included.

As the intervention group (i.e. those receiving integrated HCV treatment) were expected to be the same as those attending the OAT clinic, data regarding HCV treatment were applied within the OAT clinic setting. In the integrated arm, HCV-related investigations and treatment were offered within the OAT clinics, meanwhile, in the standard-of-care arm, HCV-related investigations and treatment were referred to and undertaken in the hospital setting, which was the infectious diseases department in Bergen.

Cost assessments

A mixed approach was used to estimate the costs of HCV treatment for this study from a health provider perspective. For capital costs, we used a gross costing top-down approach to estimate the total capital costs.

For clinical costs for the integrated and standard-of-care arms, an activity-based bottom-up approach was used to estimate the total and average costs per patient per year. These costs were estimated using the main INTRO-HCV trial data set, combined with the OAT data set from 2017 to estimate any changes by activity of the personnel type for intervention group-allocation of activity related to integrated treatment. It was assumed that there were no changes to activity percentage by each personnel from year 2017 to 2019.

Cost inputs: Variables costs and fixed costs

Cost components were divided into (A) variable costs and (B) fixed costs. See the table below for details of activities for the intervention arm and standard-of-care arm.

| HCV treatment standard-of-care arm | HCV treatment integrated (intervention) arm |
| --- | --- |
| Diagnosis HCV-consultation | Diagnosis HCV-consultation |
| Elastography | Elastography |
| Treatment HCV follow-up consultations | Treatment HCV follow-up consultations |
| Delivery of drug HCV DAA pharmacy | Delivery of drug HCV DAA pharmacy |
| Ultrasound | Delivery of drug HCV DAA |
| Arranging appointment at the hospital |  |

1. **Variable costs:**

*A1. Medication costs (DAA drug costs)*

Medication units were determined by using the patient’s medication regime for different types of HCV treatment according to their genotype status. Retail prices for ledipasvir/sofosbuvir, elbasvir/ grazoprevir, sofosbuvir/velpatasvir, glekaprevir, pibrentasvir, sofosbuvir, velpatasvir, voxilaprevir, and ribavirin were obtained from the Norwegian Medicines Agency (Legemiddelverket). Prices are for 2020.

To calculate the unit cost of a medication, the retail price was divided into the units included in the medication pack. Total direct medication costs for treatment of HCV were calculated by multiplying the number of times medication was taken per HCV treatment regimen with their respective unit costs. The cost was adjusted for the patients who have dropped out from the study by only using the amount of medication they have received before dropping out.

*A2. Personnel costs, including administration & support personnel costs (HR costs, consultations)*

To estimate the personnel costs, allocation of units and costs was done for each activity (see above table). To allocate costs for these units, average annual salaries for 2017, 2018, and 2019 were obtained from the Norwegian Central Statistics Bureau for each type of staff (e.g. resident doctor, specialist doctor, nurse, bioengineer, medical secretary, pharmacist). Then, the hourly cost for each staff was calculated by taking 48 weeks and 40 hours worked weekly per year. Average time spent (minutes) on each activity was acquired from the staff at the clinic and hospital, and these were adjusted to the hourly time spent.

To identify the unit cost per delivery by each staff, the hourly salary was multiplied with the hourly time spent for each activity. The total cost of clinical activity per year calculated by multiplying the number of different types of activity for each staff type at the clinic with their respective unit costs. These costs were calculated separately by year over the three years from 2017 to 2019, but were not adjusted for inflation here.

HR costs were estimated by calculating personnel costs for administration and support personnel, including health provider expenses, and using their contribution percentage to the OAT clinic.

*A3. Pharmacy delivery.*

A unit cost of supervised delivery for methadone and buprenorphine (for provision of OAT) from pharmacies was set to $4 and $11 (2020 US dollars) in 2017 by the Norwegian Health Ministry. However, the price for delivery of HCV treatment for pharmacies is not known. Instead, we have calculated the unit cost of delivering HCV medications for pharmacy personnel by assuming that the time used is the same as for delivery of medication in the OAT clinic.

*A4. Laboratory costs*

The unit cost of laboratory analysis regarding HCV serology was obtained from Bergen University Hospital. Then, the total number of patients each year for both intervention and standard-of-care arm were multiplied by its respective unit costs to acquire laboratory costs.

1. **Fixed costs:**

*B1. Training costs*

These costs were calculated by multiplying the number of hours spent on monthly training with the cost per hour for each personnel type.

*B2. Elastography/ultrasound*

Diverse costs such as the elastography machine (interview-based) and ultrasound were taken from online sources (<https://lbnmedical.com/ultrasound-price-guide/>) and confirmed by interviewing related personnel. Machine costs were calculated once over 2017-2019.

*B3. Building/infrastructure*

The capital costs in 2017 included rent, office equipment, and maintenance of the buildings, which were acquired retrospectively from the administrative financing database (OKRA) for LAR Bergen. For the standard-of-care arm for INTRO-HCV, a small percentage (5%) of building/infrastructure costs for the infectious diseases department was assumed to account for activities related to HCV treatment. For integrated treatment in the clinic, we did not include the building/infrastructure costs. Building costs for the standard-of-care arm were adjusted for inflation for the 3-year period (2017/2018/2019)

Initial distribution of participants by health state

The number of participants in each initial health state were obtained from the INTRO-HCV trial.^1^ Mild (METAVIR F0/F1) and moderate fibrosis (METAVIR F2/F3) states could not be distinguished, so patients with mild fibrosis were assumed to be evenly distributed across METAVIR F0 and F1 and, similarly, patients with moderate fibrosis were assumed to be evenly distributed across METAVIR F2 and F3. Alternative assumptions on initial distribution of HCV disease states are explored in sensitivity analyses. Uncertainty was incorporated by sampling the proportion in each health state using a Dirichlet distribution with the mean proportions being the point estimates from the INTRO-HCV trial.

Determining treatment rates

In the INTRO-HCV trial, the proportion treated differed under the two pathways. In the integrated treatment pathway, it was observed that 98.0% initiated treatment in total (after two years) with 93.9% starting within the first year; meanwhile, in the standard-of-care treatment pathway, 77.3% initiated treatment with 72.0% starting within the first year.^1^ The Kaplan-Meier plots of these treatment initiation data suggested that, under each pathway, the treatment coverage or total possible proportion that could be treated appeared to be approaching an asymptote or upper bound limit. An intuitive explanation of this difference in total treated proportions in the integrated pathway versus the standard-of-care pathway is that continuing forward in time, there would be an upper limit on the total proportion utilising treatment services depending on the pathway, and so some people would not manage to engage in treatment, resulting in a proportion left untreated. In this analysis, it was assumed that patients in either treatment pathway who did not engage in treatment during the two years of the INTRO-HCV trial were not likely to engage in subsequent years (we explore varied assumptions on treatment duration in sensitivity analyses). The standard-of-care pathway involved separate appointments and referrals for substance use disorders, other medical conditions, and HCV assessment and treatment, likely at different locations, with travel costs incurred by the patient. Meanwhile, the integrated pathway adopted a simplified integrated approach where frequent follow-up appointments occurred in the same physical location (either OAT clinic or CCC) and covered not only substance use disorders but also other medical conditions and HCV treatment. This synergy reduces the need for travelling, requiring fewer blood samples taken, and enabling regular contact with staff that are familiar to the patients. These components suggest a higher cumulative coverage of HCV treatment in the integrated pathway compared to the standard-of-care pathway.

In the first year, the treated proportion is taken directly from the first-year trial outcomes (integrated: 139/148 = 0.939; standard: 108/150 = 0.720). In the second or any subsequent year, the treated proportion is the ratio of the treatments given in that year divided by the remaining number of people that are not treated in the previous year. For simplicity, we assume that, following the first year, the annual background treatment transition probability (i.e. annual background treated proportion) for each pathway is derived from the treated proportion in the second year (integrated: 6/9 = 0.667; standard: 8/42 = 0.191). Both the first-year treatment rate and the subsequent annual treatment rates are fitted and sampled from their respective Beta distributions (Supplementary Figure S1a, main text Table 1).

To determine the cumulative proportion treated after each year, we first consider the proportion that was not treated each year because these comprise the people who may then be treated in subsequent years. Let $\tau$ be the treated proportion in the first year and $\hat{\tau}$ be the annual background treated proportion after the first year. The total proportion not treated after year $n$ is given by the expression $\left( 1-\tau\right)\left( 1-\hat{\tau} \right)^{n-1}$, hence the total proportion that is treated after year $n$ is

$$f\left( \tau,\hat{\tau},n \right)=\left[ 1-\left( 1-\tau\right)\left( 1-\hat{\tau} \right)^{n-1} \right]$$

For example, the proportion treated after year 1 is $f\left( 1 \right)=\tau$ and after year 2 is $f\left( 2 \right)=1-(1-\tau)(1-\hat{\tau})$. These mimic the estimated cumulative treated proportion that was observed from the INTRO-HCV trial outcomes assuming only static elements (Supplementary Figure S1b). In the base case analysis, we assume only first-year and second-year treatment occurs.

Adjustment of higher proportion of HCV genotype 3

Infection with HCV genotype 3 (G3) is associated with heightened risk of disease progression, namely, a 1.30 (95%CI 1.22-1.39) times increased relative risk of developing compensated cirrhosis (assumed to be the same for decompensated cirrhosis) and a 1.80 (95%CI 1.61-2.03) times relative risk of developing HCC.^2^ Transition probabilities for HCV disease progression derived from the literature (Table 1) were based on clinical cohorts consisting of primarily of HCV genotype 1 or other non-G3 HCV genotypes (67-100%), with a lower representation of HCV G3 (assumed to be 25%). These baseline transition probabilities were then adjusted to account for the higher proportion of HCV G3 in the INTRO-HCV cohort (approximately 60%) and among HCV infections more generally in Norway.

Non-adjustment of SMR for HCV-related death

In our analysis, we have not adjusted the standardised mortality ratio (SMR) of PWID in Western Europe specifically to exclude HCV-related mortality (see main text Table 1). However, the effect of HCV mortality is likely to be small compared to other competing causes of death for PWID. This assumption is supported by mortality data from the VIDUS and ACCESS cohorts which found that the death rate due to liver disease amongst their PWID cohort was only 2.1 per 1000 person-years overall and contributed only 6% of deaths in HCV-positive PWID.^3,4^ For this reason, the SMR for PWID in Western Europe was therefore not adjusted.

Utility index values from trial data

Health-related quality of life health (HRQoL) measures were collected from the INTRO-HCV trial^1^ using a generic questionnaire (EuroQoL EQ-5D-5L)^5^ among patients with chronic HCV infection at baseline prior to treatment initiation and at follow-up one year after the baseline assessment for successful treatment. The EQ-5D-5L instrument consists of two parts, of which the descriptive part was included in this study. It evaluates health in five dimensions; mobility, usual activities, pain/discomfort, and anxiety/depression, where each dimension has five levels of response ranging from “no problems” to “extreme problems”. The EQ-5D-5L instrument has been used to capture health state variation related to chronic HCV, including disease progression states, and cure or SVR across a range of global settings, with a systematic literature review and meta-analysis published in 2019 demonstrating that clinically important HCV disease stages are associated with differences in HRQoL as measured by the EQ-5D questionnaire.^6^

Responses to the five dimensions were than coded as single-digit numbers, which represent a numerical description of a health state. Altogether 3125 possible health states can be defined from the EQ-5D-5L descriptive part. Since the digits have no arithmetic properties, an appropriate value set which is a representative sample of the general population is required to derive the summary index score into an index value. Moreover, the EQ-5D-5L summary index is derived by using a formula that assigns values (also called weights or utilities) to each of the levels in each dimension, and calculated by deducting the corresponding weights from the value set, 1 (full health) to 0 (dead), with negative values indicating health states worse than death. In this study, HRQoL measures were stratified by fibrosis stages corresponding to METAVIR F0-F4 stages and converted to the corresponding health utilities using a UK value set in the absence of a Norwegian value set.

There are two main reasons for using a UK value set: Firstly, when the population norms for the EQ-5D were established for the Norwegian population in 2018, a UK value set was used.^7^ Secondly, the EQ-5D serves as a health care quality indicator, which includes the National Health Service for England and Wales Patient Reported Outcomes Measures (PROMs) programme and Norwegian National Quality Registries (NQR), for which it is the most widely used PROM. The Norwegian Medicines Agency (NMA) not only recommends the use of EQ-5D in all technology assessments^8^, in the absence of a Norwegian value set and scoring algorithm, the NMA also recommends the EQ-5D-3L algorithm for the UK, together with a mapping algorithm.^9,10^ This is because the overall demographics and the health systems are believed to be not very different between the UK and Norway. We followed the NMA recommendations for the HRQoL data and used the UK value set to compare the results of our cohort with the general Norwegian population as well as in the INTRO-HCV trial.

Individual-level HRQoL utility index values were available from the INTRO-HCV trial for 289 out of 298 participants. Of these 289 participants, 235 participants had data on the severity of liver fibrosis, which was measured as elastography scores in kPa using FibroScan, while 54 participants had missing elastography scores. Therefore, this yielded a total of 235 participants with data on fibrosis, as well as utility index values prior to treatment and at one-year follow-up, with missing HRQoL values in the latter being imputed using the former values. The HRQoL data were split accordingly by fibrosis stages, by translating elastography scores to METAVIR F0-F4 stages, assuming that kPa ≤ 7 corresponds to mild fibrosis (F0-F1) (n=150), 7 < kPa ≤ 12 corresponds to moderate fibrosis (F2-F3) (n=48), and kPa > 12 corresponds to compensated cirrhosis (F4) (n=37). Uncertainty distributions were determined by sampling from triangular distributions with the peak at the median and lower/upper bounds from the inter-quartile range (IQR).

Applying health utilities

We estimated the impact of treatment for each pathway in terms of total quality-adjusted life-years (QALYs) gained by applying the health utilities to their respective HCV disease progression states and accounting for current injecting status by taking the minimum of the health utilities for current injecting status and the respective disease progression stage, which would reflect the quality of life associated with the worse health state of two overlapping health conditions, as has been assumed in other HCV cost-effectiveness modelling studies.^11^ For example, for someone who is currently injecting (health utility = 0.73) and is HCV-infected with compensated cirrhosis (health utility = 0.63), we would assign a health utility of 0.63. The total QALYs in this population are calculated annually and accumulated over the time horizon. Although the model is not explicitly stratified by injecting status, health utilities are time-varying input parameters that change following injecting cessation. Specifically, for each model run, the health utilities for not currently injecting drugs are applied after the injecting duration has passed for each model run.

Univariate sensitivity analysis and comparison of different settings

Details of univariate sensitivity analysis scenarios undertaken are described in Supplementary Table S3. We also undertook sensitivity analyses to estimate how cost-effectiveness of integrated treatment versus standard-of-care treatment varies depending on assumptions on the costs of delivering HCV treatment and providing healthcare management of HCV-associated disease (scenarios S1-S4). In these scenario analyses, we assumed that HCV delivery was the same as our Norway model (scenario S0), but used corresponding costs from country-specific settings as case examples (namely, the UK, US, France, and Australia) to highlight the differences in cost assumptions (see main text Table 3 and Supplementary Table S4). These scenarios are: (S1) similar treatment costs but lower healthcare management costs for late-stage HCV-related disease (using costs from the United Kingdom); (S2) higher treatment costs (using costs from the United States); (S3) lower treatment costs but higher healthcare costs (using costs from France); and (S4) lower costs of both treatment and healthcare management (using costs from Australia). The costs that were used for each scenario came from published studies in the countries reflecting the scenarios.

For the multi-setting analysis, the total treatment unit costs per patient were assumed to be the same for both standard and integrated care – this yielded a conservative estimate since our costing exercise for INTRO-HCV revealed integrated treatment was slightly less expensive than standard-of-care treatment. Specifically, DAA medication costs, non-DAA treatment costs, and annual healthcare costs for managing HCV-related disease were obtained from published cost-effectiveness studies for each country (Supplementary Table S4). Actual negotiated DAA prices were confidential but were likely to be much lower than the list price so, for these analyses, we have considered DAA medication list prices as well as 30%/60%/90% reductions from the DAA medication list prices. Meanwhile, annual healthcare costs for managing HCV-related disease were adjusted by producer price indices (PPI) to the year 2021.

Point estimates and uncertainty distributions for the various cost components in the different scenarios or settings (which corresponded to other countries where we took the cost data) are presented in local currency in Supplementary Table S4 and converted to 2021 Euros using purchasing power parities (PPP) currency conversion rates (see main text Table 3 for the converted cost assumptions in 2021 Euros).

Supplementary Results

Total costs and QALYs

Without treatment, the disease burden was estimated to incur healthcare costs of €14,400 (95%UI 8,300-23,300) per person that was diagnosed for managing HCV-related disease in this population over 50-years (Supplementary Table S5). Implementing HCV treatment incurred treatment costs, but reduced healthcare costs. For instance, the standard-of-care treatment pathway reduced healthcare costs to €9,700 (95%UI 5,500-15,500) per diagnosed person, while also incurring treatment costs of €21,100 (95%UI 16,600-26,000) per diagnosed person, for a total cost of €31,100 (95%UI 24,300-38,800) per diagnosed person. Meanwhile, the integrated treatment pathway was estimated to lead to even lower healthcare costs of €8,100 (95%UI 4,800-13,100) per diagnosed person, while incurring higher treatment costs of €25,900 (95%UI 20,900-30,800) per diagnosed person, for a total cost of €34,000 (95%UI 27,100-41,600) per diagnosed person, over the 50-year time horizon (Supplementary Table S5). Compared to the standard-of-care pathway, the integrated pathway increased costs €2,900 (95%UI 900-5,600) per diagnosed person, at DAA list price, with this reducing to €1,600 (95%UI 0-3,100) per diagnosed person, or €210 (95%UI -1,200 to 1,300) per person, for a 30% or 60% reduction in DAA drug costs, respectively.

With respect to health impact, overall, the integrated pathway was associated with an additional 0.2 (95%UI 0.1-0.5) QALYs gained per diagnosed person compared to the standard-of-care pathway over the 50-year time horizon.

Treatment uptake and cascade-of-care

Compared to the standard-of-care pathway, the integrated pathway resulted in a 23.0% (95%UI 11.7-37.4%) relative increase in the number of PWID initiating treatments including re-treatments (integrated 298.9 [95%UI 288.7-310.0] versus standard 244.3 [95%UI 218.6-265.9]) and a 32.0% (95%UI 19.3-51.3%) relative increase in the number of PWID achieving SVR (integrated 276.3 [95%UI 263.6-282.6] versus standard 208.4 [95%UI 182.6-230.1]) over the 50-year time horizon (Supplementary Figure S3a, Supplementary Table S6).

Disease burden

In the counterfactual scenario without treatment, the model estimated that there would be 132.3 (95%UI 86.3-187.8) new cases of compensated cirrhosis, 102.3 (95%UI 48.6-172.4) new cases of ESLD, and 71.8 (95%UI 31.2-133.9 HCV-related deaths over the 50-year time horizon (Supplementary Figure S3b, Supplementary Table S7). Compared to no treatment, the standard-of-care treatment pathway would avert approximately 40% of these new ESLD cases and HCV-related deaths, with the integrated treatment pathway averting an additional 12-13% (Supplementary Figure S3b, Supplementary Table S7).

Reductions in DAA prices improve cost-effectiveness substantially

The cost of DAA medications had a strong effect on the ICER and the probability of integrated treatment pathway being cost-effective and cost-saving (main text Figure 3, Supplementary Figure S5). Our model results suggest that a 30% reduction in DAA medication cost (€17,800 for 12-weeks) was associated with an ICER of €6,910 per QALY gained with a 99.2% and 91.4% probability of being cost-effective at the conventional and lower WTP thresholds, respectively, and a 2.6% probability of being cost-saving. A 60% reduction in DAA medication cost (€10,200 for 12-weeks) had a lower ICER of €929 per QALY gained with at least a 99% probability of being cost-effective at either WTP threshold, and a 36.1% probability of being cost-saving. Meanwhile, a 90% reduction in DAA medication cost (€2,500 for 12-weeks) was cost-saving with a negative ICER of -€5,010 per QALY gained and a 99.6% probability of being cost-saving. The cost-effectiveness plane and cost-effectiveness acceptability curves (CEAC) at baseline and for the specific reductions in DAA drug price (30%/60%/90%) are shown in Supplementary Figure S4 and main text Figure 2, respectively.

The estimated DAA medication prices needed to have a 50%, 80%, or 99% probability of being cost-effective at either WTP threshold or cost-saving are shown in Supplementary Figure S6. For example, our model results suggest that a DAA medication cost of €22,000 (13.4% reduction from baseline) and €11,100 (56.3% reduction from baseline) would be needed for the integrated treatment pathway to have an 80% and 99% probability, respectively, of being cost-effective at the lower WTP threshold. A relatively similar but still lower DAA medication cost of €8,600 (66.1% reduction) would be associated with a 50% probability of being cost-saving. Meanwhile, cheaper DAA medication costs of €6,400 (74.8% reduction from baseline) and €2,900 (88.6% reduction) would be needed for integrated treatment to have an 80% or 99% probability of being cost-saving, respectively (Supplementary Figure S6).

Supplementary Figures


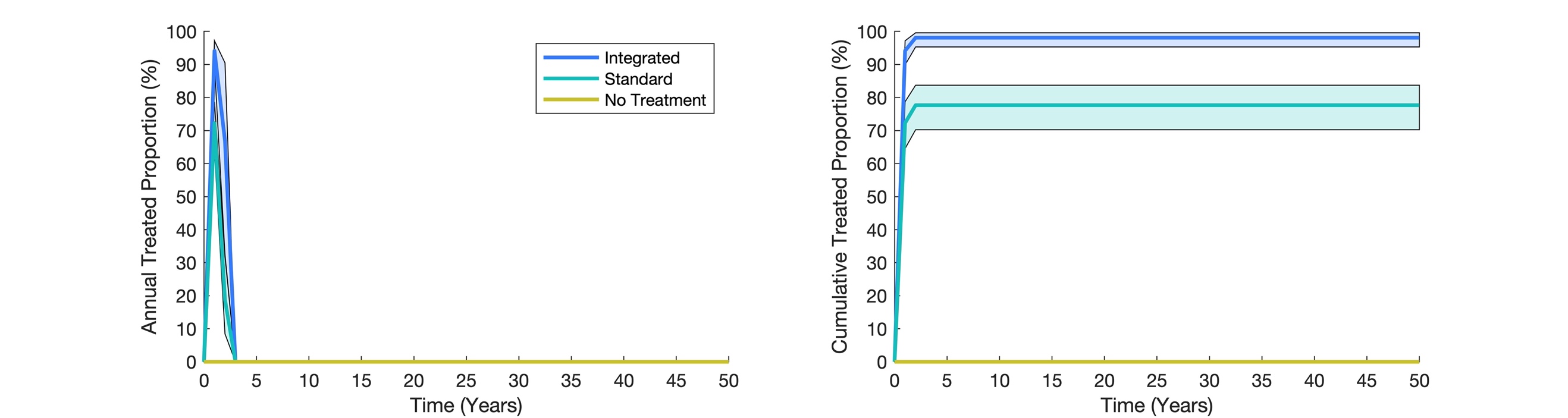


Supplementary Figure S1. Model input parameters for background treatment rate.

(A) Annual background treated proportions derived from the INTRO-HCV trial that are used as model input parameters. (B) Estimated cumulative treated proportion assuming only static elements (i.e. with no re-infections nor deaths included).


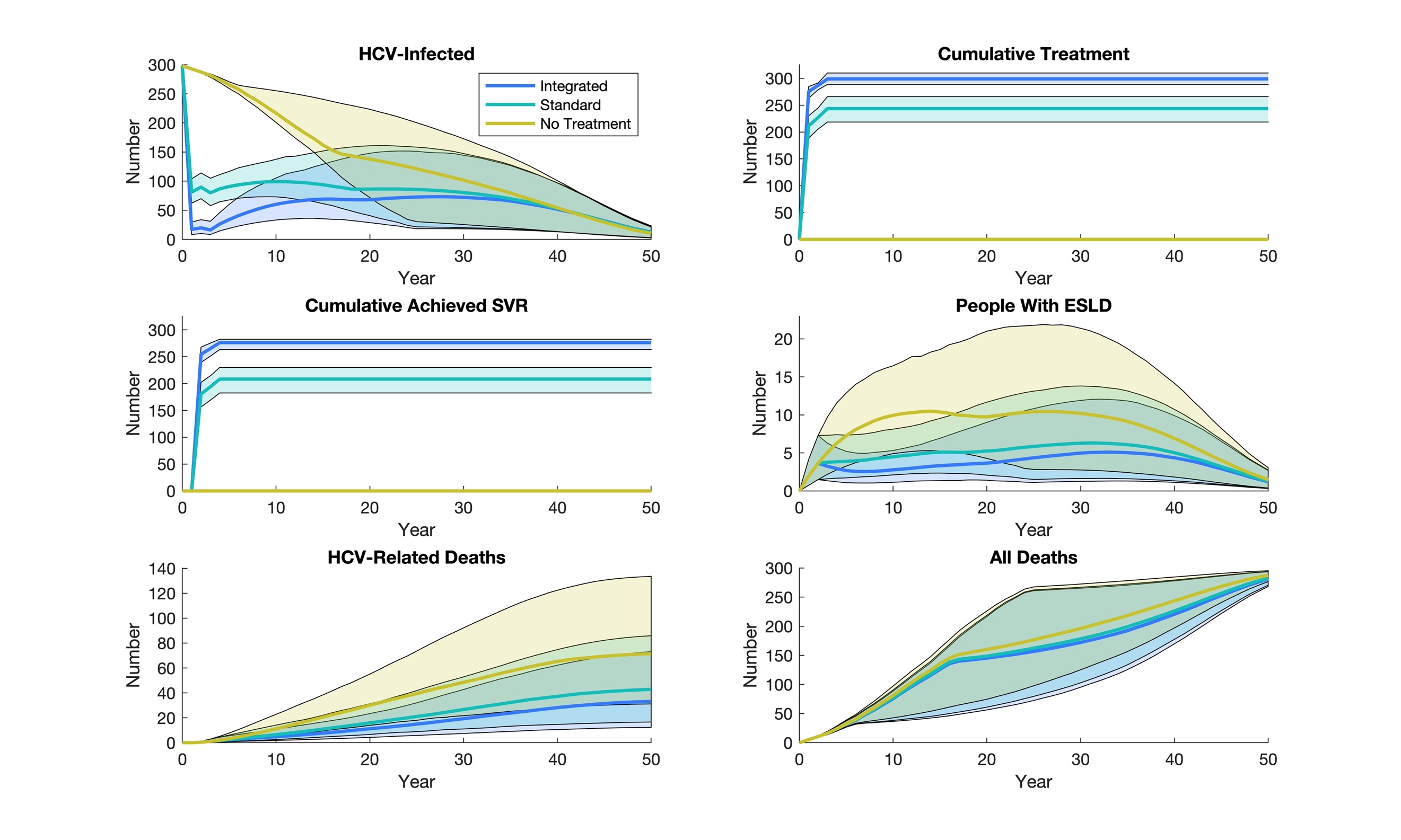


Supplementary Figure S2. Selected modelled health states over time.

Time series plots showing the number of patients in various health states, as well the number of HCV-related or all-cause deaths, in any given year over the 50-year time horizon for the integrated treatment pathway, standard-of-care treatment pathway, and no treatment scenarios. Results are shown as the median and 95% uncertainty interval of 1,000 model simulations.

| **(A)** |
| --- |
| 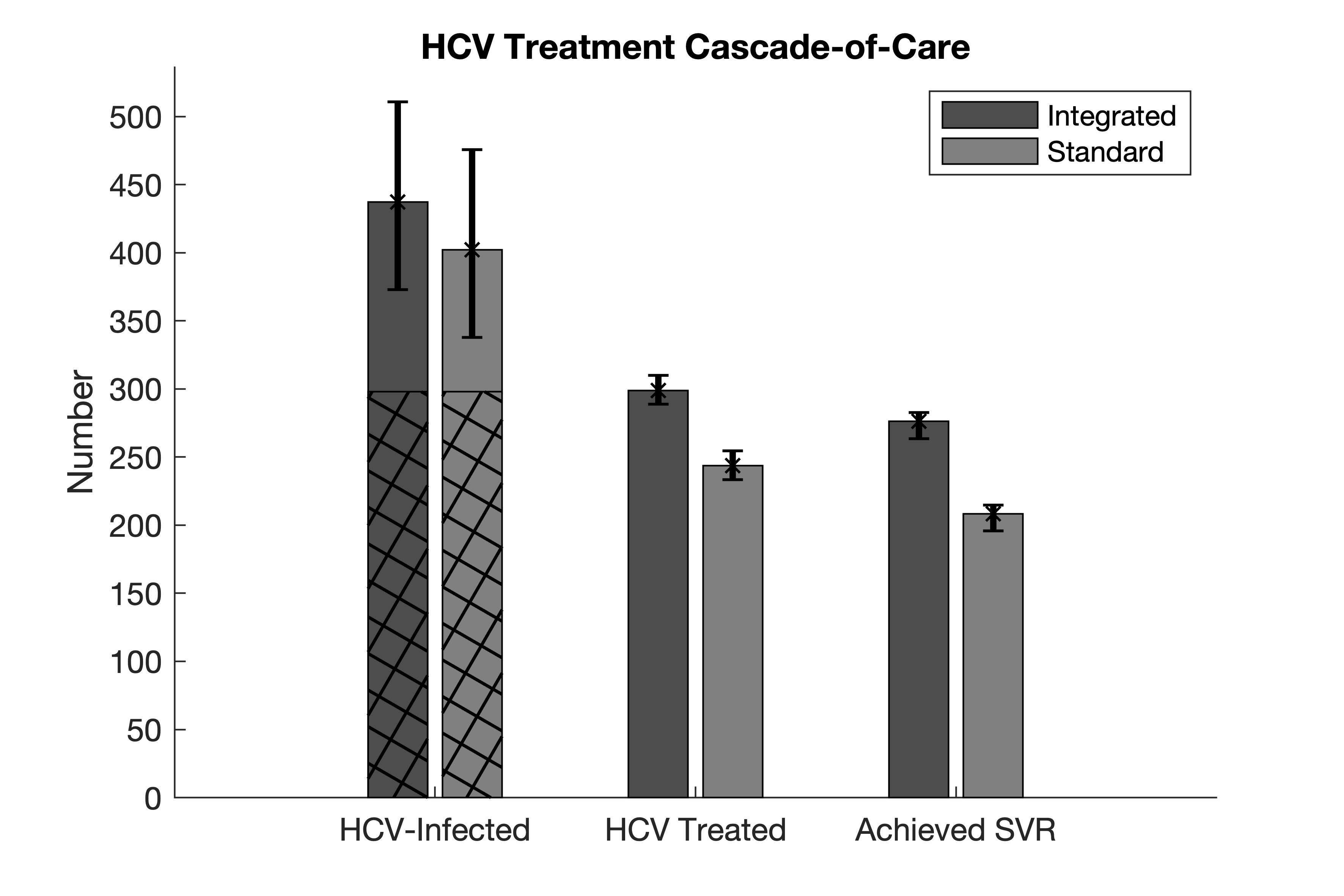 |
| **(B)** |
| 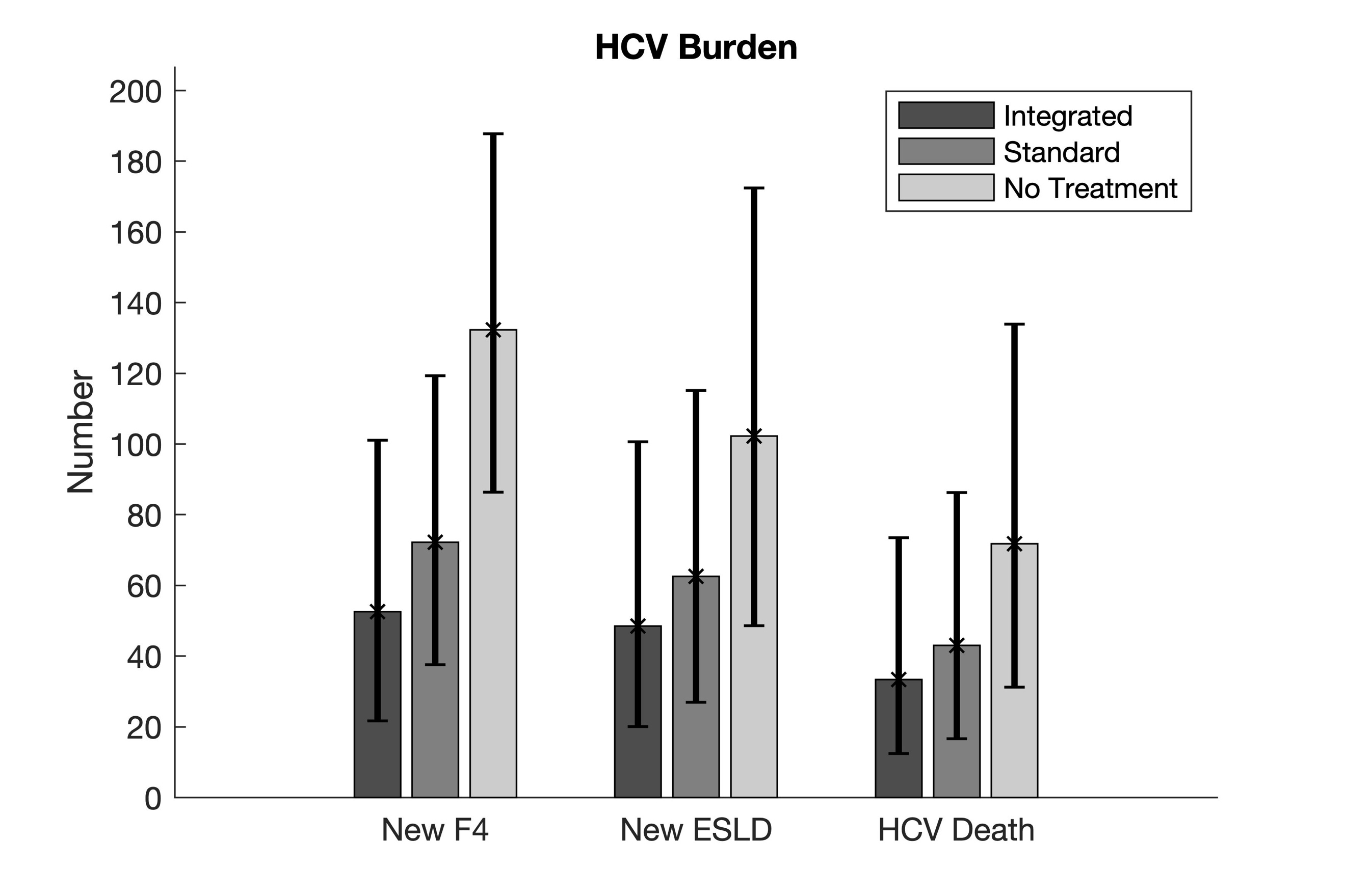 |

Supplementary Figure S3. HCV cascade of care and HCV burden.

Bar graphs showing (A) the HCV treatment cascade-of-care for the integrated and standard-of-care treatment pathways; and (B) the projected cumulative case numbers of compensated cirrhosis (F4/CC), end-stage liver disease (DC or HCC), and HCV-associated deaths for the integrated and standard-of-care pathways, and if no treatment had occurred. Hatching indicates the number of initial HCV-infected persons, whereas the full bar includes cumulative re-infections. Time horizon is 50 years. Results are shown as the median and 95% uncertainty interval of 1,000 model simulations. See also Supplementary Tables S6 and S7.

**
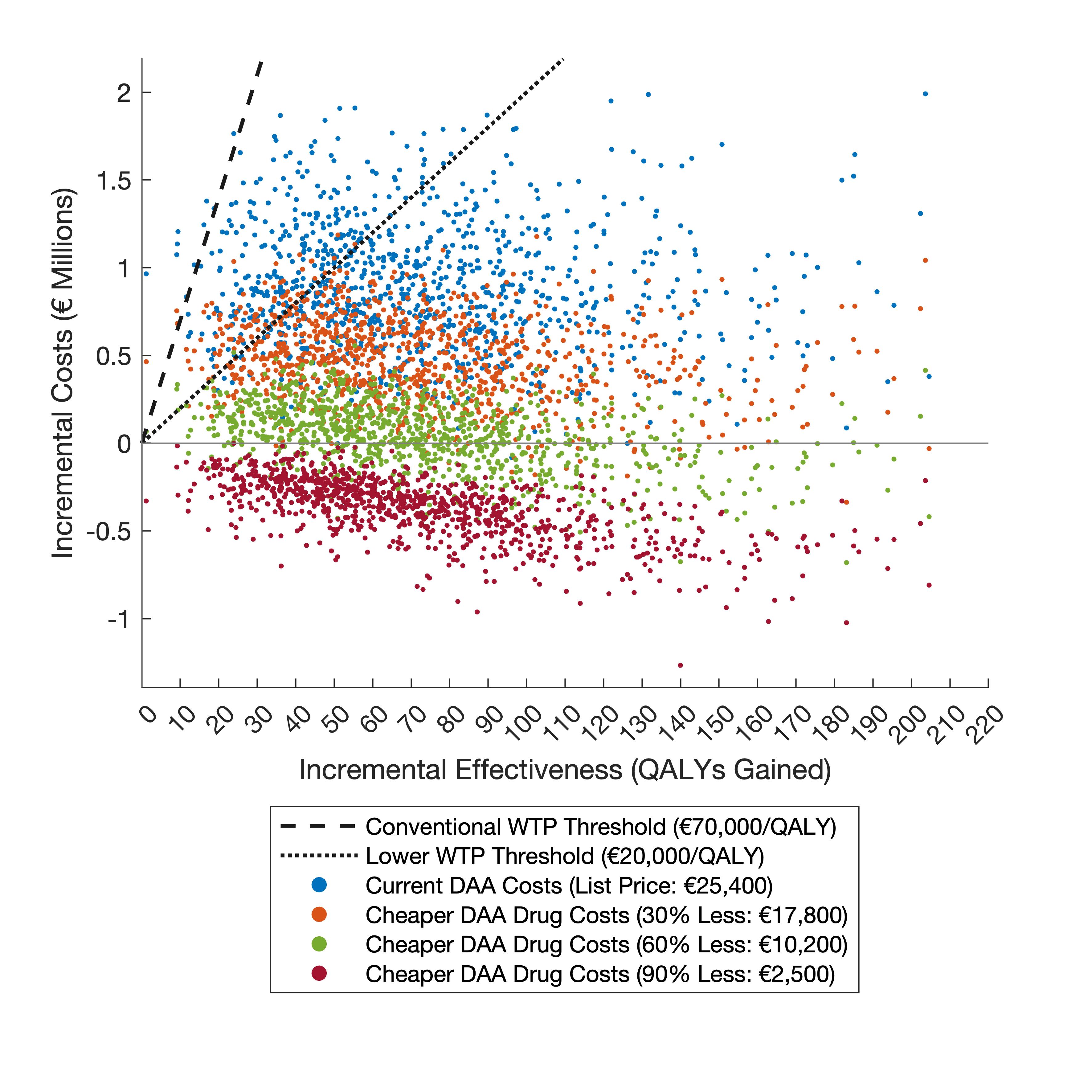
**

Supplementary Figure S4. Cost-effectiveness plane at baseline and selected reductions in DAA price.

Probabilistic sensitivity analysis of the modelled results for the integrated treatment pathway compared to the standard-of-care treatment pathway from the INTRO-HCV intervention trial with respect to the cost-effectiveness plane showing the incremental costs (vertical axis) plotted against the incremental quality-adjusted life year (QALYs) gained (horizontal axis). The plots are shown for the baseline scenario using DAA medication list price as well as for selected reductions in DAA medication prices of 30%/60%/90%. Costs and QALYs are discounted at a rate of 4.0% per annum. Time horizon is 50 years. The conventional (€70,000/QALY, dashed line) and lower (€20,000/QALY dotted line) willingness-to-pay (WTP) thresholds are shown. Results are for 1,000 model simulations.


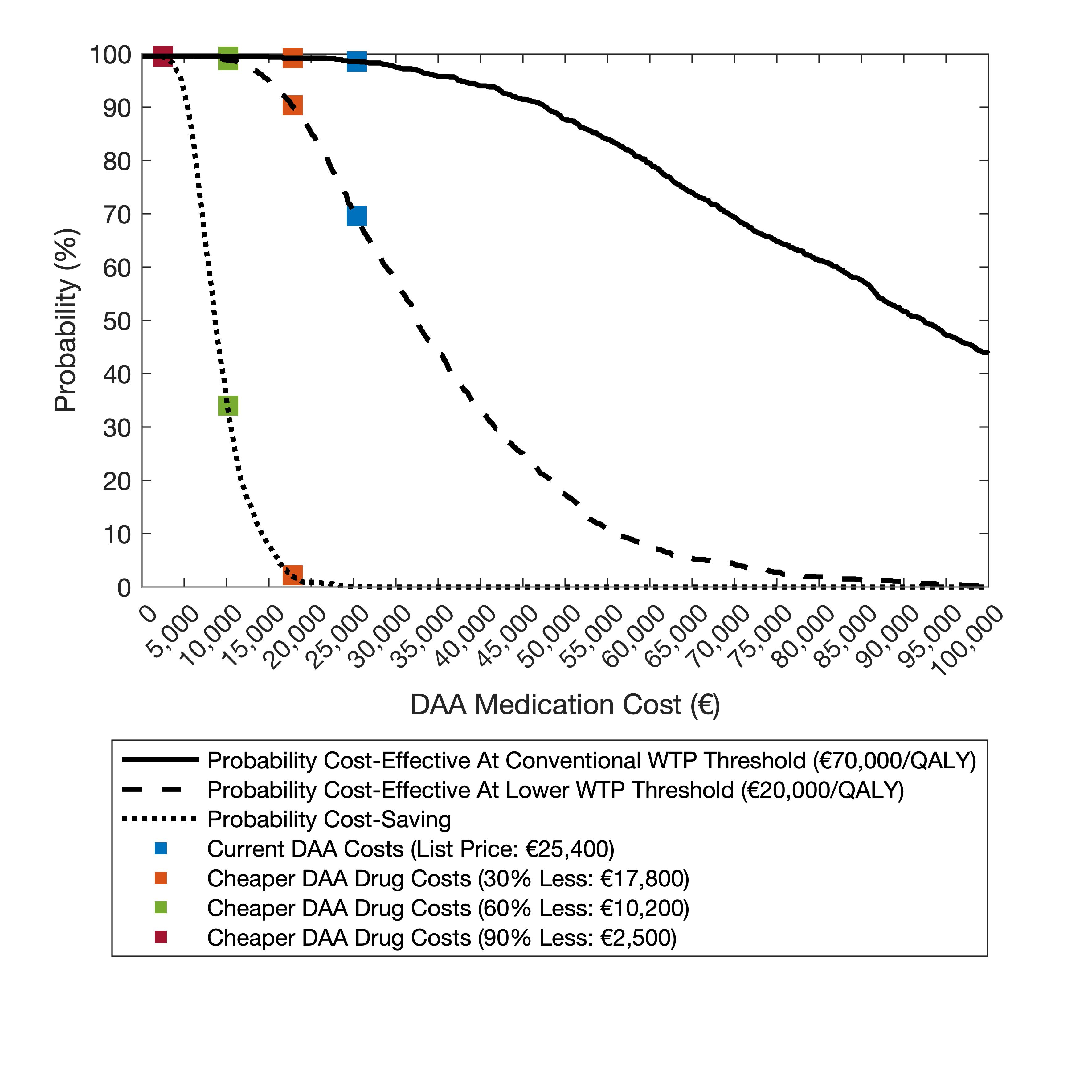


Supplementary Figure S5. Effect of DAA medication price on probability of cost-effectiveness.

Probabilistic sensitivity analyses showing the effects of varying the price of DAA medications on the probability of being cost-effective at conventional (€70,000/QALY) and lower (€20,000/QALY) willingness-to-pay thresholds and of being cost-saving for the integrated treatment pathway compared to standard-of-care treatment pathway. Costs and QALYs are discounted at a rate of 4.0% per annum. Time horizon is 50 years. Results are for 1,000 model simulations.


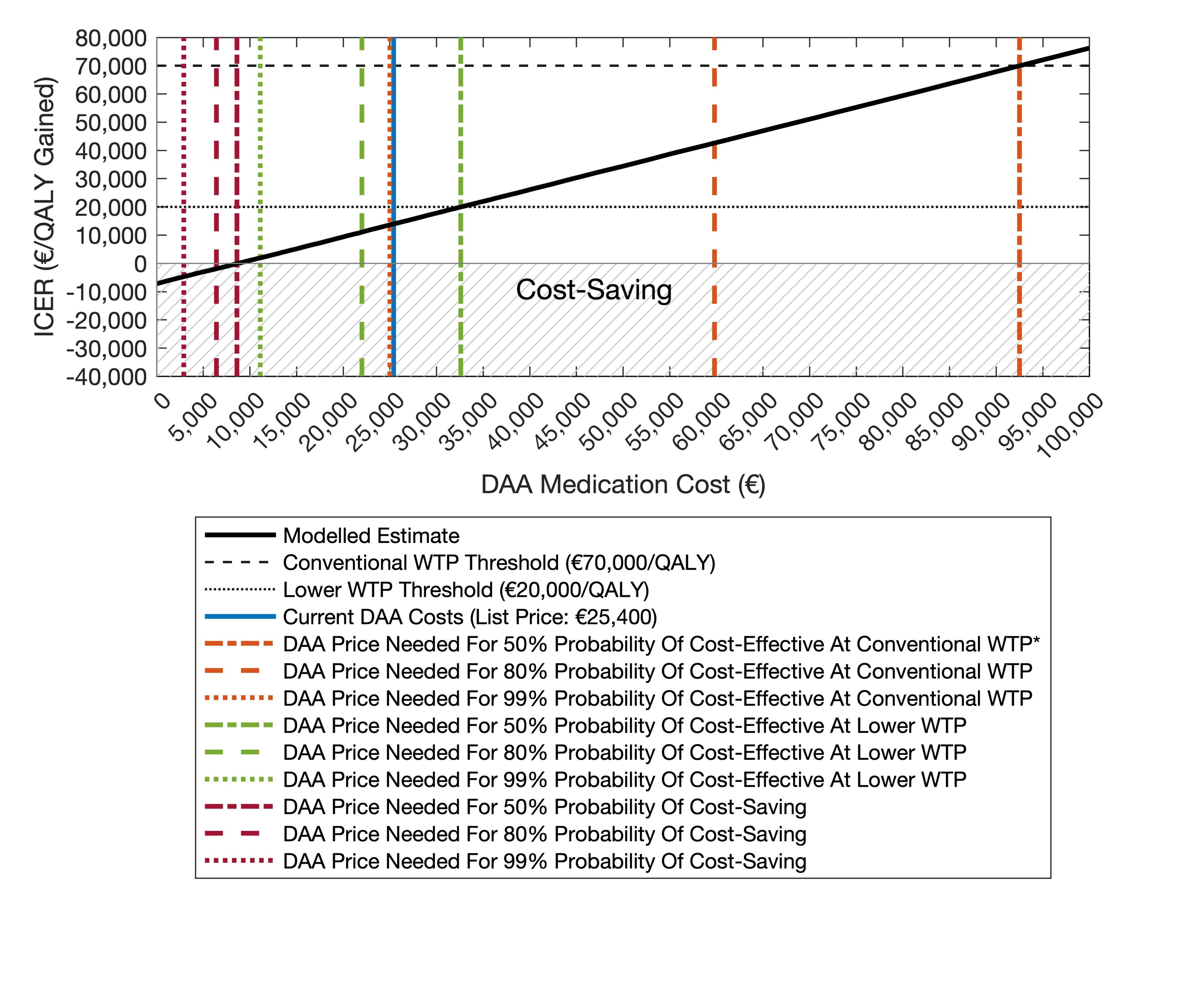


Supplementary Figure S6. ICER by varying DAA medication cost.

Probabilistic sensitivity analysis showing the direct relationship between the incremental cost-effectiveness ratio (ICER) of the integrated treatment pathway compared to the standard-of-care treatment pathway, and the DAA medication cost. Costs and QALYs are discounted at a rate of 4.0% per annum. Time horizon is 50 years. Results are for 1,000 model simulations. Areas with hatching indicate cost-savings and a positive health impact, and so where integrated treatment is dominant. *Note: In our analysis, we have varied DAA price to a maximum of €100,000. A DAA price of €100,000 is likely to have a greater than 50% probability of being cost-effective at the conventional WTP threshold.


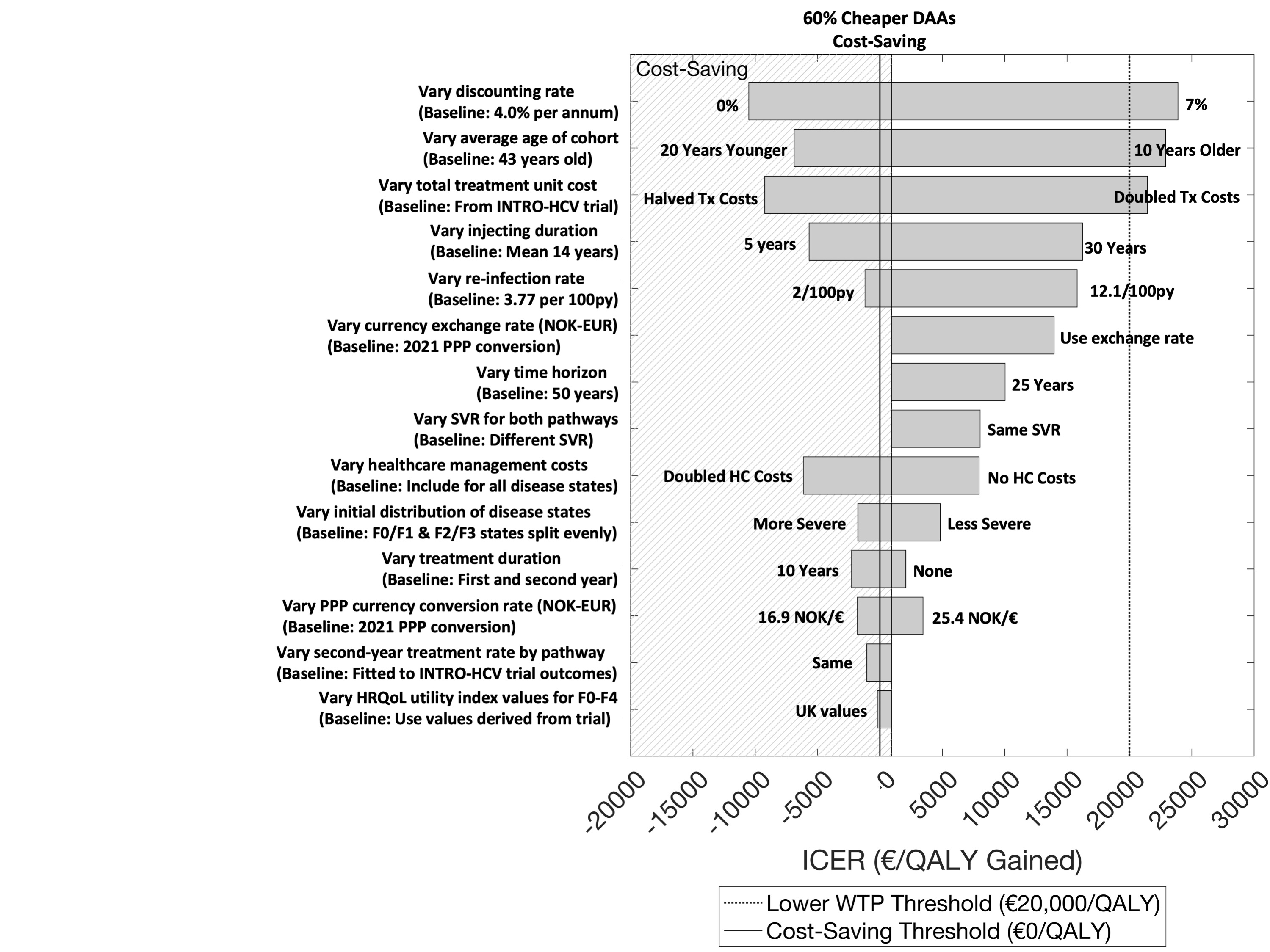


Supplementary Figure S7. Univariate sensitivity analyses with 60% DAA price reduction.

Univariate sensitivity analyses on the incremental cost-effectiveness ratio (ICER) of the integrated treatment pathway compared to the standard-of-care treatment pathway, assuming a 60% DAA price reduction across all scenarios (i.e. €10,200 for a 12-week treatment course). The scenarios presented here are placed in the same order as the univariate sensitivity analysis in the main text (Figure 4) using DAAs at list price for ease of comparison (note that the scale of the horizontal axis is different from Figure 4). Costs and QALYs are discounted at a rate of 4.0% per annum. Time horizon is 50 years. The lower (dotted line) willingness-to-pay (WTP) and cost-saving (solid line) thresholds are shown. Results are for 1,000 model simulations. Areas with hatching indicate cost-savings and a positive health impact, and so where integrated treatment is dominant.

| **(A)** Probability of integrated treatment being cost-effective at a conventional €70,000/QALY WTP threshold for Norway. |
| --- |
| 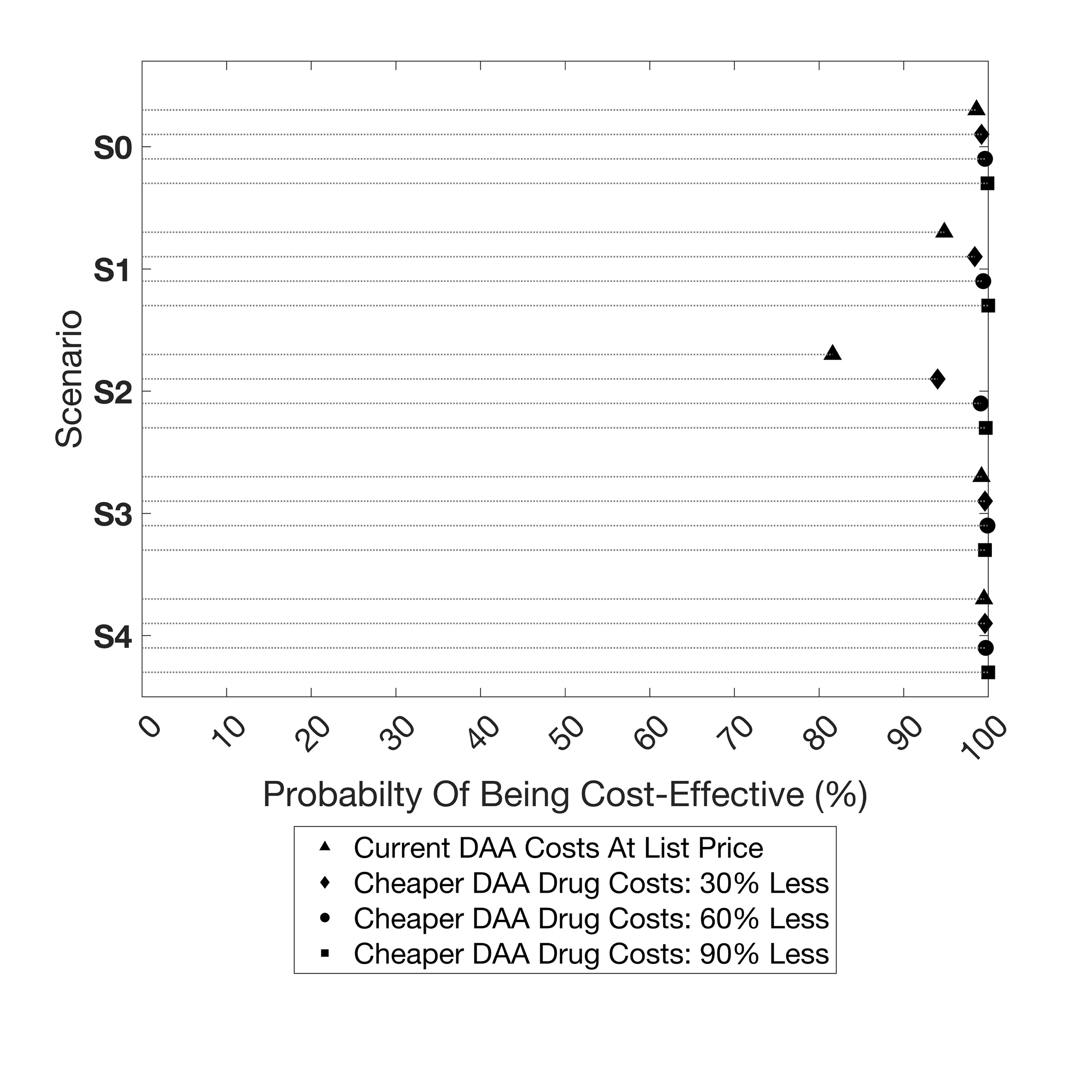 |
| **(B)** Probability of integrated treatment being cost-effective at a lower €20,000/QALY WTP threshold. |
| 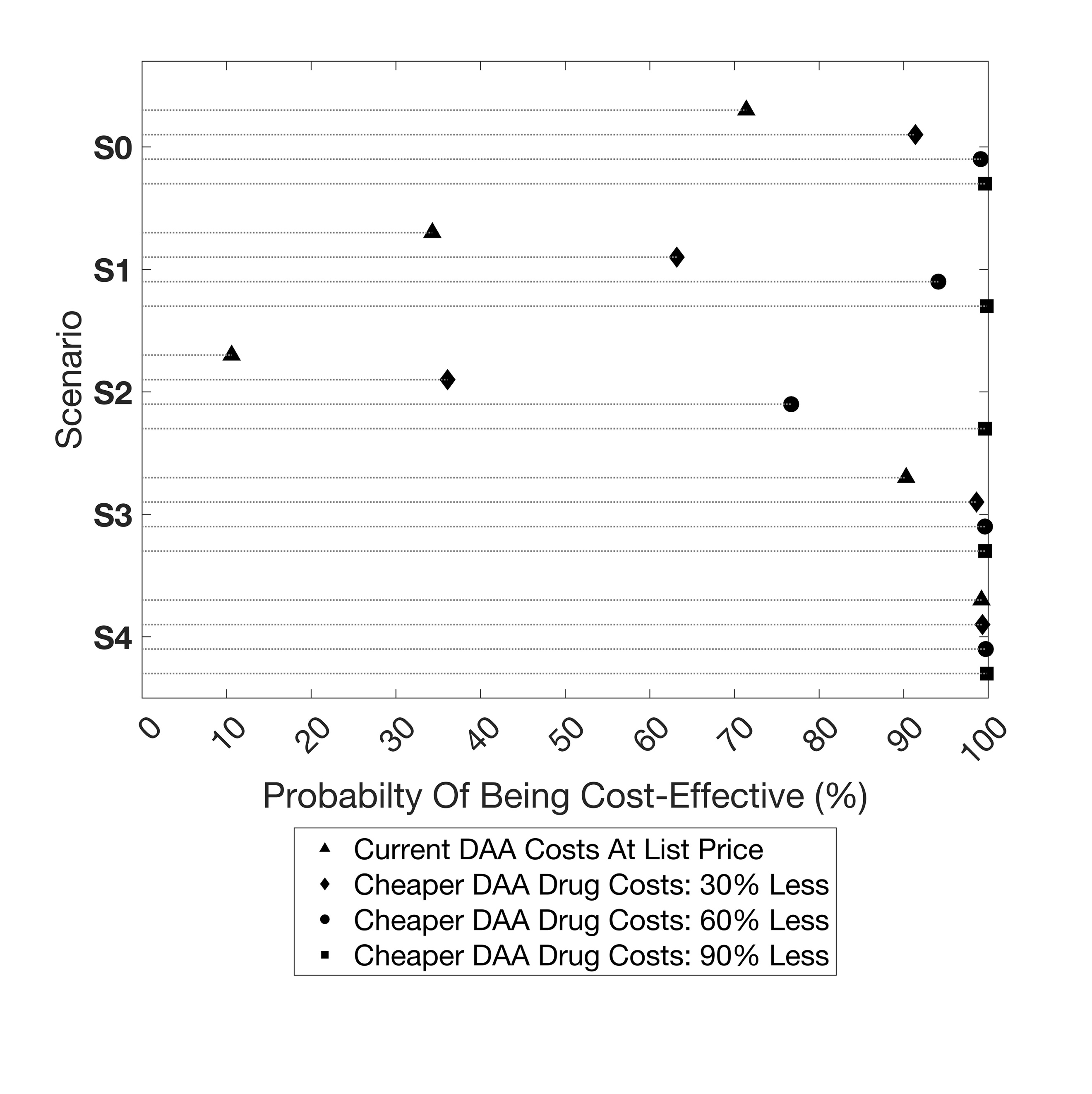 |
| **(C)** Probability of integrated treatment being cost-saving. |
| 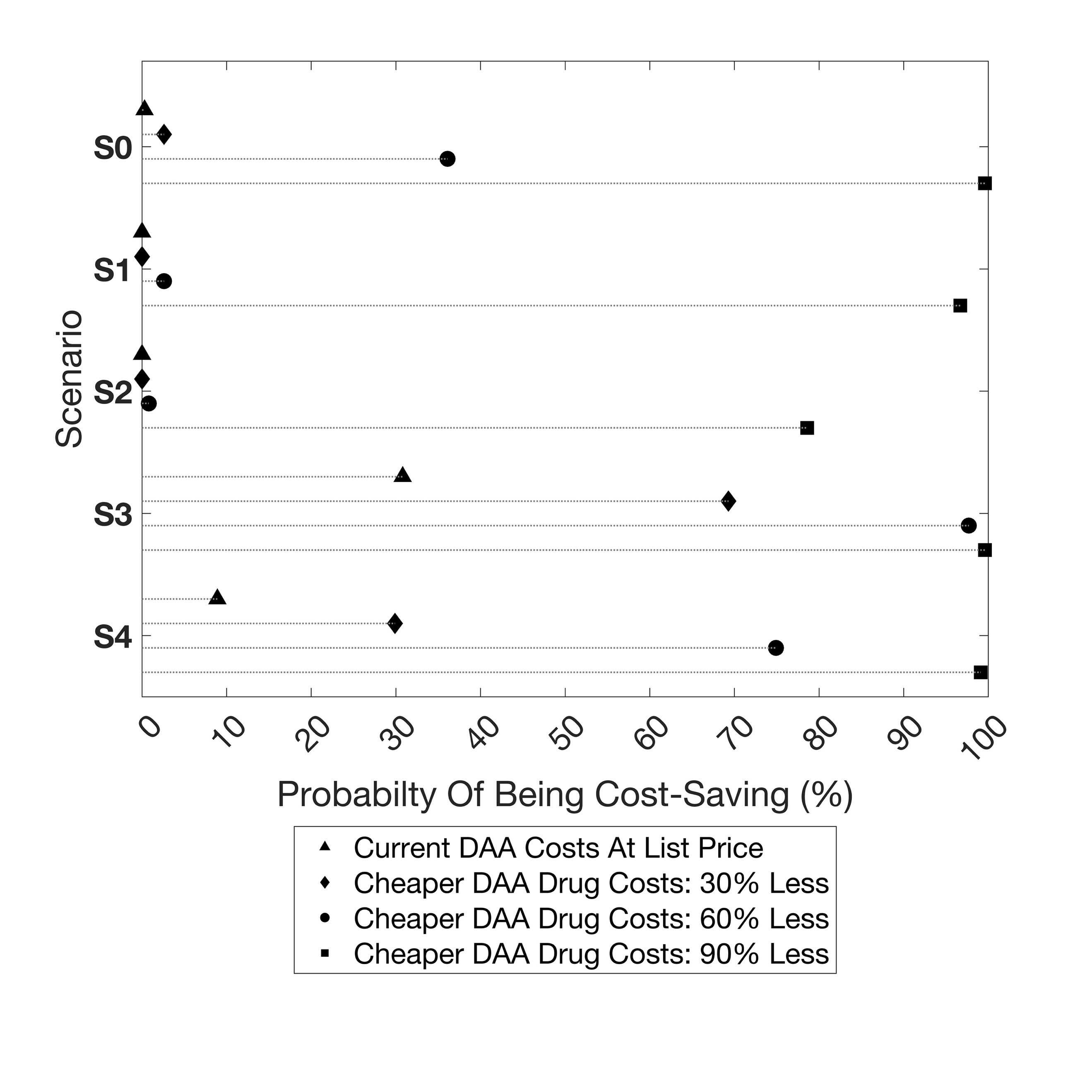 |

Supplementary Figure S8. Probability of integrated treatment being cost-effective or cost-saving.

Modelled estimates for the cost-effectiveness of delivering integrated HCV treatment compared to standard-of-care treatment as in the INTRO-HCV trial for scenarios S1-S4 representing different cost assumptions on the costs of treatment and healthcare management, and how it changes depending on the price of DAA medications, namely, DAA costs at current list prices and reductions of 30%, 60%, and 90%. These figures show the probability of integrated treatment being cost-effective at (A) conventional (€70,000/QALY) and (B) lower (€20,000/QALY) willingness-to-pay (WTP) thresholds, and (C) the probability of integrated treatment being cost-saving, compared to the standard-of-care treatment pathway. Non-DAA-related HCV treatment costs and healthcare management costs are adjusted to 2021 for inflation by producer price index (PPI), with total treatment unit costs converted to 2021 Euros using purchasing power parities (PPP) for cross-setting comparison. Costs and QALYs are discounted at a rate of 4.0% per annum. Time horizon is 50 years. Results are for 1,000 model simulations.

Supplementary Tables

Supplementary Table S1. Annual health state transition probabilities and initial conditions.

Annual transition probabilities and initial distribution of HCV-related health states used in the model. DC: decompensated cirrhosis; HCC: hepatocellular carcinoma; LT: liver transplant; PLT: post-liver transplant; PSA: probabilistic sensitivity analysis.

|  | | **Baseline or fitted value/range, distribution for PSA** | **Source/comment** |
| --- | --- | --- | --- |
| **Annual transition probabilities†** | | | |
| F0 to F1 | | 0.109 (95%CI 0.071-0.168), Normal | Stage-specific fibrosis progression rates among PWID obtained from a systematic review and meta-analysis of fibrosis progression^12^ |
| F1 to F2 | | 0.071 (95%CI 0.052-0.096), Normal |  |
| F2 to F3 | | 0.121 (95%CI 0.086-0.170), Normal |  |
| F3 to F4 (i.e. CC) | | 0.194 (95%CI 0.135-0.278), Normal |  |
| Hazard ratio of disease progression if infected by HCV genotype 3† | | F4 to DC: 1.30 (range 1.22-1.39), Uniform  F4 or DC to HCC: 1.80 (range 1.60-2.03), Uniform | Adjustment factor for heightened disease progression associated with HCV genotype 3.^2^ |
| F4 to DC† | | 0.039 ($\alpha=14.6168, \beta=360.1732$), Beta | ^13^ |
| Hazard ratio for F4 to DC if SVR | | 0.07 (95%CI 0.03, 0.20), Lognormal | ^14^ |
| F4 or DC to HCC† | | 0.014 ($\alpha=1.9326, \beta=136.1074$), Beta | ^13^ |
| Hazard ratio for F4 to HCC if SVR | | 0.23 (95% CI 0.16, 0.35), Lognormal | ^14,15^ |
| Hazard ratio for DC to HCC if SVR | | 1.0 | Assume same progression for both SVR and non-SVR |
| DC or HCC to LT | | 0.03 ($\alpha=6.5256, \beta=210.9945$), Beta | ^13^ |
| LT to post-LT | | Derived from complement of LT-related death | Assume those who have not died following LT move to post-LT. |
| DC-related death | | 0.13 ($\alpha=147.03, \beta=983.97$), Beta | ^13^ |
| HCC-related death | | 0.43 ($\alpha=117.1033, \beta=155.23$), Beta | ^13^ |
| LT-related death | | 0.21 ($\alpha=16.2762, \beta=61.2294$), Beta | ^13^ |
| Post-LT-related death | | 0.057 ($\alpha=2.902, \beta=378.8825$), Beta | ^13^ |
| **Initial distribution by HCV disease progression** | | | |
|  | F0-F1 | 201 | The number of participants in each initial health state were obtained from the INTRO-HCV trial. Uncertainty was incorporated by sampling the proportion in each health state using a Dirichlet distribution with the mean proportions being the point estimates from the INTRO-HCV trial. |
|  | F2-F3 | 62 |  |
|  | F4 | 35 |  |
|  | DC | -- |  |
|  | HCC | -- |  |
|  | LT | -- |  |
|  | Post-LT | -- |  |
|  | Total | 298 |  |

†These transition probabilities are adjusted to reflect the higher proportion in the INTRO-HCV trial of HCV genotype 3 (G3=60%), which is associated with an increased transition probability of disease progression.

Supplementary Table S2. HCV treatment costs and annual healthcare costs in 2021 NOK.

Total treatment costs for HCV, including DAA drug costs, non-DAA variables costs, and fixed costs were obtained from the INTRO-HCV trial to estimate the total treatment unit costs per patient. Annual healthcare costs managing HCV-related disease were estimated for this study using hospital records from an outpatient clinic (OAT Laksevåg). Costs from the study were originally derived in 2020 NOK, with non-DAA costs inflated to 2021 and presented below in 2021 NOK. Meanwhile, converted costs to 2021 Euros as used in the model are shown in main text Table 2 (using the purchasing power parity (PPP) currency conversion rate of 1 EUR = 20.3 NOK for 2021). DC: decompensated cirrhosis; HCC: hepatocellular carcinoma; LT: liver transplant; NA: not applicable; PLT: post-liver transplant.

|  | | **Integrated** | **Standard** | **Source/comment** |
| --- | --- | --- | --- | --- |
| **Treatment costs per patient** | | | | |
| DAA drug costs (12-week) | | NOK 516,458.97 | NOK 520,085.62 | Variable cost of DAA medications only. The main DAA regimens used throughout the INTRO-HCV trial period were elbasvir/grazoprevir, sofosbuvir/ledipasvir, or sofosbuvir/velpatasvir, with SVR assessed at 12 weeks after completion of DAAs irrespective of treatment pathway. |
| Non-DAA variable costs | |  |  | Variable costs other than DAA drugs, broken down by costs of human resources (HR), consultations, pharmacy delivery, and laboratory tests. |
|  | HR costs, consultations | NOK 1,619.84 | NOK 950.55 |  |
|  | Pharmacy delivery cost | NOK 11.30 | NOK 70.45 |  |
|  | Laboratory tests | NOK 1,198.56 | NOK 1,198.56 |  |
| Fixed costs | |  |  | Fixed costs, broken down by costs of training, elastography/ultrasound, and building/infrastructure. |
|  | Training costs | NOK 756.41 | NOK 359.77 |  |
|  | Elastography/ultrasound | NOK 3,739.64 | NOK 6,947.55 |  |
|  | Building/infrastructure | NA | NOK 1,361.66 |  |
| Total treatment unit costs* (per patient) | | NOK 523,784.72 | NOK 530,974.15 | Sum of DAA drug costs, non-DAA variable costs, and fixed costs. |
| **Annual healthcare costs* for managing HCV-related disease** | | | | |
|  | F0-F1 | NOK 3,908 | |  |
|  | F2-F3 | NOK 3,908 | |  |
|  | F4 | NOK 10,381 | |  |
|  | DC | NOK 291,748 | |  |
|  | HCC | NOK 407,890 | |  |
|  | LT (transplantation) | NOK 1,339,545 | | One-time cost of liver transplantation |
|  | LT | NOK 161,297 | | Hospital/healthcare management costs incurred in year of liver transplantation |
|  | PLT | NOK 161,297 | |  |

*Total treatment unit cost per patient and all annual healthcare management costs have a +/-20% uncertainty associated with them and are each sampled from their respective uniform distributions.

Supplementary Table S3. Details of univariate sensitivity analyses undertaken.

DC: decompensated cirrhosis; HCC: hepatocellular carcinoma; LT: liver transplant; PLT: post-liver transplant.

| **Sensitivity Analysis Scenario** | **Description** |
| --- | --- |
| **X1.** Vary total treatment unit cost | Assume half or double the total treatment unit cost at baseline. |
| **X2.** Vary re-infection rate | Assume lower re-infection incidence of 2 per 100 person-years (py) or higher re-infection incidence of 12.1 per 100py compared to the baseline re-infection rate (3.77 per 100py). The lower re-infection incidence (2/100py) is based on the annual incidence needed to achieve WHO HCV elimination targets for incidence among PWID^16^, whilst the higher re-infection incidence (12.1/100py) is based on possible high re-infection rate among PWID occurring in community pharmacies in Tayside, Scotland (which provide services to PWID including OAT provision).^17^ We assumed a linear transition (i.e. decrease or increase) between the baseline re-infection rate and the varied re-infection rate occurring over the first 10 years, stabilising thereafter. |
| **X3.** Vary second-year treatment rate | Assume the second-year treatment rate for the integrated pathway is the same as for the standard pathway (19.1%), compared to the integrated pathway having a higher second-year treatment rate at baseline (66.7%). |
| **X4.** Vary treatment duration | Assume no treatment after the first year or continued treatment rate at the same rate as the second year over a period of 10 years. |
| **X5.** Vary average age of cohort | Assume average age of cohort is 20 years younger or 10 years older than at baseline (43 years old). |
| **X6.** Vary initial distribution of disease states | Assume the initial distribution of disease states is less severe (all F0/F1 are F0 and F2/F3 are F2) or more severe (all F0/F1 are F1 and F2/F3 are F3), compared to baseline (F0/F1 are split evenly between F0 and F1; similarly, F2/F3 are split evenly between F2 and F3). |
| **X7.** Vary injecting duration | Assume injecting duration across the cohort is 5 years or 30 years compared to baseline (14 years). |
| **X8.** Vary healthcare management costs | Assume no healthcare management costs, or double healthcare management costs for all disease states, including one-time liver transplantation costs compared to baseline. |
| **X9.** Same SVR for both pathways | Assume that SVR is the same for both integrated and standard-of-care treatment pathways, compared to baseline where SVR differs as observed in the INTRO-HCV trial. In this scenario, SVR rates for both pathways were sampled from uniform distributions with range spanning the mean SVRs from the trial. Specifically, SVR: 0.911 (range: 0.875-0.946), Uniform. |
| **X10.** Vary HRQoL utility index values | Assume that all HRQoL utility index values, including for METAVIR stages F0 to F4, are derived from UK-based values published in the literature.^13,18^ Note that at baseline, utility index values for METAVIR stages F0 to F4 were derived from the INTRO-HCV trial data. Specifically, assume that:  F0-F1: Infected: 0.77 (Beta; $\alpha=521.2375, \beta=155.6943$), SVR: 0.82 (Beta; $\alpha=65.8678, \beta=14.4588$);  F2-F3: Infected: 0.66 (Beta; $\alpha=168.2461, \beta=86.6723$), SVR: 0.72 (Beta; $\alpha=58.0608, \beta=37.1124$);  F4: Infected: 0.55 (Beta; $\alpha=47.1021, \beta=38.5381$), SVR: 0.61 (Beta; $\alpha=58.0608, \beta=37.1124$);  DC: 0.45 (Beta; $\alpha=123.75, \beta=151.25$); HCC: 0.45 (Beta; $\alpha=123.75, \beta=151.25$);  LT: 0.45 (Beta; $\alpha=123.75, \beta=151.25$); PLT: 0.67 (Beta; $\alpha=59.2548, \beta=29.1852$) |
| **X11.** Vary time horizon | Assume time horizon of 25 years compared to baseline (50 years). |
| **X12.** Vary discounting rate | Assume no (0%) or double (8%) the baseline annual discounting rate (4%) applied to costs and outcomes (QALYs). |
| **X13.** Vary PPP currency conversion rate | Assume currency conversion rates using PPP are 20% in favour of the Euro (i.e. 1 NOK = 0.0394 EUR or 25.4 NOK per EUR) or against the Euro (i.e. 1 NOK = 0.0591 EUR or 16.9 NOK per EUR), based on variation in the PPP conversion rates over the past 10 years. |
| **X14.** Vary currency exchange rate | Assume currency conversion using market-based exchange rates averaged over 2021 (1 NOK = 0.0984 EUR or 10.1597 NOK per EUR), as derived from estimates published by OECD: (<https://data.oecd.org/conversion/exchange-rates.htm>). |

Supplementary Table S4. Parameter estimates and uncertainty for the various cost components in selected countries.

Mean costs used in sensitivity analyses estimating the cost-effectiveness of integrated versus standard-of-care treatment in settings with different conditions, using the costs from selected countries as sources of these scenarios. Costs for HCV treatment and annual healthcare management for HCV-related disease were obtained from published estimates corresponding to each setting. All costs are presented in local currency for each country, and converted to 2021 Euros using purchasing power parities (PPP) to undertake the cost-effectiveness analyses within each setting. The converted costs in 2020 Euros are shown in main text Table 3. DC: decompensated cirrhosis; HCC: hepatocellular carcinoma; LT: liver transplant; PLT: post-liver transplant; PPI: producer price index. N/A: not applicable.

|  | | **Scenario S0**  **(Norway/NOK)** | **Scenario S1**  **(UK/£)** | **Scenario S2**  **(US/USD)** | **Scenario S3**  **(France/€)** | **Scenario S4**  **(Australia/AUD)** | **Source/comment** |
| --- | --- | --- | --- | --- | --- | --- | --- |
| **Treatment costs per patient** | | | | | | | |
| DAA drug costs (12-week) | | | | | | | |
|  | DAA list price | 516,459 | 38,988 | 85,500^a^ | 28,730 | 13,190 | DAA list prices obtained from selected country studies representing each scenario: UK^19^, US^20^, France^21^, Australia^22,23^. For the sensitivity analyses, DAA medication costs were reduced by 30%/60%/90% from the published list price. |
|  | 30% reduction | 361,521 | 27,292 | 59,850 | 20,111 | 9,233 |  |
|  | 60% reduction | 206,584 | 15,595 | 34,200 | 11,492 | 5,276 |  |
|  | 90% reduction | 51,646 | 3,899 | 8,550 | 2,873 | 1,319 |  |
| Other HCV-related treatment costs**†** | | | | | | | |
|  | | Itg: 7,326  [6,827 $\times$ PPI’_A_]  Std: 10,889  [10,147 $\times$ PPI’_A_] | 416  [394 $\times$ PPI’_B_] | 3,200  [2,592 $\times$ PPI’_C_] | 790  [740 $\times$ PPI’_D_] | 1,978  [1,846 $\times$ PPI’_E_] | Other HCV-related treatment costs besides DAA drugs, including non-DAA variable costs (e.g. HR, consultations, pharmacy delivery, laboratory tests) and fixed costs (e.g. training, elastography/ ultrasound, building/ infrastructure), adjusted to 2021 using PPI. |
| Total treatment unit costs (per patient) | | | | | | | |
|  | DAA list price | 523,785 | 39,404 | 88,700 | 29,520 | 15,168 | Sum of DAA drug costs for specified price reductions, and other treatment costs. |
|  | 30% reduction | 368,847 | 27,708 | 63,050 | 20,901 | 11,211 |  |
|  | 60% reduction | 213,909 | 16,011 | 37,400 | 12,282 | 7,254 |  |
|  | 90% reduction | 58,972 | 4,315 | 11,750 | 3,663 | 3,297 |  |
| **Annual healthcare costs† for managing HCV-related disease (in original currency)** | | | | | | | |
|  | F0-F1 | 3,908  [3,642 $\times$ PPI_A_] | 213  [138 $\times$ PPI_B_,  (Gamma; $k$=25·6995, $\theta$=5·3698) $\times$ PPI_B_] | 914  [728 $\times$ PPI_C_,  (Gamma; $k$=15.37, $\theta$=47.37) $\times$ PPI_C_] | 810  [$738\times$ PPI_D_] | 489  [447 $\times$ PPI_E_] | Annual healthcare costs for managing HCV-related disease came from published cost-effectiveness studies conducted for UK^13,24^, US^25,26^, France^21,27^, Australia^22^. Uncertainty distributions were used if available, otherwise point estimated were used. |
|  | F2-F3 | 3,908  [3,642 $\times$ PPI_A_] | 1,106  [717 $\times$ PPI_B_,  (Gamma; $k$=88·8502, $\theta$=8·0698) $\times$ PPI_B_] | 925  [$737\times$ PPI_C_,  (Gamma; $k$=15.37, $\theta$=47.98) $\times$ PPI_C_] | 910  [$829\times$ PPI_D_] | 489  [447 $\times$ PPI_E_] | Note: For US and Australia, assumed costs of F2-F3 from F2 costs |
|  | F4 | 10,381  [9,674 $\times$ PPI_A_] | 1,756  [1,138 $\times$ PPI_B_, (Gamma; $k$=24·2342, $\theta$=46·9584) $\times$ PPI_B_] | 2,190  [1,745 $\times$ PPI_C_, (Gamma; $k$=15.37, $\theta$=113.59) $\times$ PPI_C_] | 8,828  [$8044\times$ PPI_D_] | 1,023  [935 $\times$ PPI_E_] |  |
|  | DC | 291,748  [271,870 $\times$ PPI_A_] | 14,069  [9,120 $\times$ PPI_B_, (Gamma; $k$=36·0249, $\theta$=253·1582) $\times$ PPI_B_] | 24,336  [19,389 $\times$ PPI_C_, (Gamma; $k$=15.37, $\theta$=1261.79) $\times$ PPI_C_] | 28,631  [$26,089\times$ PPI_D_] | 16,638  [15,202 $\times$ PPI_E_] |  |
|  | HCC | 407,890  [380,099 $\times$ PPI_A_] | 12,537  [8,127 $\times$ PPI_B_, (Gamma; $k$=18·1081, $\theta$=448·8045) $\times$ PPI_B_] | 44,753  [35,655 $\times$ PPI_C_, (Gamma; $k$=15.37, $\theta$=2320.34) $\times$ PPI_C_] | 31,259  [$28,484\times$ PPI_D_] | 11,776  [10,760 $\times$ PPI_E_] |  |
|  | LT (transplantation) | 1,339,545  [1,248,278 $\times$ PPI_A_] | 42,161  [27,330 $\times$ PPI_B_, (Gamma; $k$=89·7536, $\theta$=304·5004) $\times$ PPI_B_] | 129,409  [103,102 $\times$ PPI_C_, (Gamma; $k$=15.37, $\theta$=6703.71) $\times$ PPI_C_] | 161,135  [$146,829\times$ PPI_D_] | 159,308  [145,565 $\times$ PPI_E_] | One-time cost of liver transplantation |
|  | LT | 161,297  [150,307 $\times$ PPI_A_] | 14,591  [9,458 $\times$ PPI_B_, (Gamma; $k$=13·7788, $\theta$=686·4168) $\times$ PPI_B_] | * | * | * | Hospital/healthcare management costs incurred in year of liver transplantation |
|  | PLT | 161,297  [150,307 $\times$ PPI_A_] | 2,137  [1,385 $\times$ PPI_B_, (Gamma; $k$=15·2189, $\theta$=91·0053) $\times$ PPI_B_] | 33,961  [27,057 $\times$ PPI_C_, (Gamma; $k$=15.37, $\theta$=1760.79) $\times$ PPI_C_] | 23,542  [$21,452\times$ PPI_D_] | * |  |
| **Currency conversion rates‡** | | | | | | | |
|  | Conversion to 2021 Euro using PPP | 1 EUR = 20.3000 NOK | 1 EUR = 1.0214 GBP | 1 EUR = 1.5080 USD | Currency already in EUR | 1 EUR = 2.1828 AUD | PPP conversion factors with reference to the European Union (27 countries 2020 or EU27) were derived from published data by Statistics Norway^28^ (for scenario S0) and the OECD^29^ (scenarios S1-S4). |
| **Producer price index (PPI) adjustment factors** | | | | | | | |
|  | Currency | NOK | GBP | USD | EUR | AUD |  |
|  | Year of estimates for non-DAA HCV treatment costs | 2020 | 2018 | 2016 | 2015 | 2016 |  |
|  | Year of estimates for annual healthcare costs | 2020 | 2002 | 2010 | 2010 | 2014 |  |
|  | PPI adjustment factor to 2021 | PPI’_A_ = 1.0731  PPI_A_ = 1.0731 | PPI’_B_ = 1.0560  PPI_B_ = 1.5427 | PPI’_C_ = 1.2344  PPI_C_ = 1.2552 | PPI’_D_ = 1.0681  PPI_D_ = 1.0974 | PPI’_E_ = 1.0717  PPI_E_ = 1.0944 | PPI adjustment factors derived from published reports by the OECD^29^ for scenarios S1 (UK) and S3 (France), the US Bureau of Labor Statistics^30^ for S2, and the Australian Bureau of Statistics^31^ for S4. |

*Assumed to be combined with one-time LT costs since annual costs were not available from published studies.

**†**Non-DAA-related HCV treatment costs and annual healthcare costs for chronic HCV disease stages in each country were obtained from published country-level studies and adjusted for inflation using producer price index (PPI) to 2021 from cost year in which the original study occurred, if known. In the French study, healthcare costs were obtained by taking the sum of reported ambulatory costs (i.e. out-patient costs, including never treated and after HCV treatment failure) and hospitalisation costs (i.e. in-patient costs, including no death and in-hospital death).

**‡**Currency conversion using 2021 purchasing power parities (PPP).

^a^Price of sofosbuvir/ledipasvir in the US.

Supplementary Table S5. Breakdown of total costs by treatment and healthcare management by DAA price reduction.

Breakdown of total costs per diagnosed person by treatment and healthcare management for each scenario, at baseline using DAA list price and for specific reductions in DAA price (30%/60%/90%). Costs are discounted at a rate of 4.0% per annum. Time horizon is 50 years. Total costs are divided by the total number of individuals (n=298) to report outcomes per person that was diagnosed, and are shown as the median and 95%UI of 1,000 model simulations. NA: Not Applicable.

|  | | **Treatment Costs Per Person Diagnosed**  **(€)** | **% of Total** | **Healthcare Costs Per Person Diagnosed**  **(€)** | **% of Total** | **Total Costs Per Person Diagnosed**  **(€)** |
| --- | --- | --- | --- | --- | --- | --- |
| **DAA list price (€25,400 for 12-weeks)** | | | | | | |
|  | **No Treatment** | NA | NA | 14,426  (8,276 to 23,329) | 100.0% | 14,426  (8,276 to 23,329) |
|  | **Standard** | 21,157  (16,570 to 25,992) | 68.4%  (55.8 to 79.5%) | 9,718  (5,548 to 15,498) | 31.6%  (20.5 to 44.2%) | 31,093  (24,314 to 38,756) |
|  | **Integrated** | 25,914  (20,857 to 30,847) | 76.0%  (64.4 to 84.7%) | 8,070  (4,759 to 13,144) | 24.0%  (15.3 to 35.6%) | 34,043  (27,123 to 41,601) |
| **30% DAA price reduction (€17,800 for 12-weeks)** | | | | | | |
|  | **No Treatment** | NA | NA | 14,426  (8,276 to 23,329) | 100.0% | 14,426  (8,276 to 23,329) |
|  | **Standard** | 14,985  (13,470 to 16,305) | 60.3%  (48.8 to 73.2%) | 9,718  (5,548 to 15,498 | 39.7%  (26.8 to 51.2%) | 24,548  (20,621 to 30,671) |
|  | **Integrated** | 18,136  (17,550 to 18,776) | 69.2  (57.8 to 79.2%) | 8,070  (4,759 to 13,144) | 30.8%  (20.8 to 42.2%) | 26,262  (22,912 to 31,378) |
| **60% DAA price reduction (€10,200 for 12-weeks)** | | | | | | |
|  | **No Treatment** | NA | NA | 14,426  (8,276 to 23,329) | 100.0% | 14,426  (8,276 to 23,329) |
|  | **Standard** | 8,749  (7,865 to 9,520) | 47.1%  (35.7 to 61.4%) | 9,718  (5,548 to 15,498 | 52.9%  (38.6 to 64.3%) | 18,398  (14,437 to 24,363) |
|  | **Integrated** | 10,518  (10,178 to 10,889) | 56.6%  (44.2 to 68.8%) | 8,070  (4,759 to 13,144) | 43.4% (31.2 to 55.8%) | 18,618  (15,323 to 23,756) |
| **90% DAA price reduction (€2,500 for 12-weeks)** | | | | | | |
|  | **No Treatment** | NA | NA | 14,426  (8,276 to 23,329) | 100.0% | 14,426  (8,276 to 23,329) |
|  | **Standard** | 2,514  (2,260 to 2,735) | 20.3% (13.8 to 31.4%) | 9,718  (5,548 to 15,498 | 79.7%  (68.6 to 86.2) | 12,226  (8,093 to 18,011) |
|  | **Integrated** | 2,900  (2,806 to 3,002) | 26.5%  (17.9 to 37.8) | 8,070  (4,759 to 13,144) | 73.5%  (62.2 to 82.1%) | 10,962  (7,668 to 16,099) |

Supplementary Table S6. Cascade-of-care at baseline.

Cascade-of-care for HCV treatment uptake and SVR outcomes for the integrated and standard-of-care treatment pathways.

|  | **Number Infected** | **Number Treated** | | **Number Cured/Achieved SVR** | | **Proportion Cured/Achieved SVR** | |
| --- | --- | --- | --- | --- | --- | --- | --- |
|  | **Initial** | **Total** | **Incremental** | **Total** | **Incremental** | **% of treated** | **% of all infected*** |
| **Standard** | 298 | 244.3  (218.6 to 265.9) | -- | 208.4  (182.6 to 230.1) | -- | 85.8%  (78.5 to 91.0%) | 51.6%  (44.2 to 60.4%) |
| **Integrated** | 298 | 298.9  (288.7 to 310.0) | 55.9  (31.0 to 82.6) | 276.3  (263.6 to 282.6) | 67.0  (44.3 to 93.7) | 92.3%  (88.1 to 95.1%) | 63.1%  (54.2 to 74.0%) |

*All infected includes all those who were initially infected in addition to any re-infections that occurred over the time horizon.

Supplementary Table S7. Total and incremental averted HCV-related morbidity and mortality for each scenario.

Total and incremental averted number of cases of HCV-related morbidity (F4/CC and ESLD) and mortality for each scenario.

|  | **New F4/CC** | | **New ESLD** | | **HCV-related Deaths** | |
| --- | --- | --- | --- | --- | --- | --- |
|  | **Total** | **Incremental Averted** | **Total** | **Incremental Averted** | **Total** | **Incremental Averted** |
| **No Treatment** | 132.3  (86.3 to 187.8) | -- | 102.3  (48.6 to 172.4) | -- | 71.8  (31.2 to 133.9) | -- |
| **Standard** | 72.2  (37.6 to 119.3) | 58.8  (38.3 to 90.6) | 62.5  (26.9 to 115.1) | 38.3  (19.2 to 71.7) | 43.1  (16.7 to 86.3) | 27.5  (12.2 to 54.5) |
| **Integrated** | 52.5  (21.7 to 101.0) | 17.9  (10.1 to 31.1) | 48.5  (20.0 to 100.6) | 12.3  (5.3 to 24.8) | 33.3  (12.4 to 73.4) | 9.0  (3.4 to 18.7) |

Supplementary Table S8. Incremental cost-effectiveness ratios (ICERs) at baseline per diagnosed person.

Costs and QALYs are discounted at a rate of 4.0% per annum. Time horizon is 50 years. Total and incremental costs and QALYs are divided by the total number of individuals (n=298) to report outcomes per person that was diagnosed, and are shown as the median and 95%UI of 1,000 model simulations. The probability of being cost-effective is the proportion of model simulations that are below the conventional (€70,000/QALY) and lower (€20,000/QALY) willingness-to-pay thresholds.

|  | **Costs Per Person Diagnosed (€)** | | **QALYs Per Person Diagnosed** | | **ICER** | **Probability** | **Probability** |
| --- | --- | --- | --- | --- | --- | --- | --- |
|  | **Total** | **Incremental** | **Total** | **Incremental** | **Cost/QALY Gained** | **Cost-Effective** | **Cost-Saving** |
| **No Treatment** | 14,426  (8,276 to 23,329) | -- | 8.9  (7.3 to 11.2) | -- | -- | -- | -- |
| **Standard** | 31,093  (24,314 to 38,756) | 16,359  (10,532 to 21,814) | 9.6  (7.8 to 12.2) | 0.7  (0.2 to 1.6) | 23,737 | 95.9% (Conventional)  37.9% (Lower) | 0.0% |
| **Integrated** | 34,043  (27,123 to 41,601) | 2,884  (928 to 5,562) | 9.8  (7.9 to 12.6) | 0.2  (0.1 to 0.5) | 13,272 | 98.6% (Conventional)  71.4% (Lower) | 0.3% |

Supplementary Checklist C1. Consolidated Health Economic Evaluation Reporting Standards (CHEERS) 2022.

CHEERS 2022 checklist.^32^

| **Section/item** | **Item No.** | **Guidance for reporting** | **Reported in section** |
| --- | --- | --- | --- |
| **Title** |  |  |  |
| Title | 1 | Identify the study as an economic evaluation and specify the interventions being compared. | Title |
| **Abstract** |  |  |  |
| Abstract | 2 | Provide a structured summary that highlights context, key methods, results, and alternative analyses. | Abstract |
| **Introduction** |  |  |  |
| Background and objectives | 3 | Give the context for the study, the study question, and its practical relevance for decision making in policy or practice. | Introduction, Paragraphs 1-4 |
| **Methods** |  |  |  |
| Health economic analysis plan | 4 | Indicate whether a health economic analysis plan was developed and where available. | ‘Study description’; ‘Model description’; ‘Impact and cost-effectiveness analysis’ |
| Study population | 5 | Describe characteristics of the study population (such as age range, demographics, socioeconomic, or clinical characteristics). | ‘Study description’; ‘Model description’; ‘Model parameterisation’; Table 1 |
| Setting and location | 6 | Provide relevant contextual information that may influence findings. | ‘Study description’; ‘Impact and cost-effectiveness analysis’ |
| Comparators | 7 | Describe the interventions or strategies being compared and why chosen. | ‘Study description’; ‘Impact and cost-effectiveness analysis’ |
| Perspective | 8 | State the perspective(s) adopted by the study and why chosen. | ‘Study description’; ‘Estimation of costs’; ‘Impact and cost-effectiveness analysis’ |
| Time horizon | 9 | State the time horizon for the study and why appropriate. | ‘Impact and cost-effectiveness analysis’ |
| Discount rate | 10 | Report the discount rate(s) and reason chosen. | ‘Impact and cost-effectiveness analysis’ |
| Selection of outcomes | 11 | Describe what outcomes were used as the measure(s) of benefit(s) and harm(s). | ‘Derivation of health utilities’; ‘Impact and cost-effectiveness analysis’ |
| Measurement of outcomes | 12 | Describe how outcomes used to capture benefit(s) and harm(s) were measured. | ‘Model description’; ‘Derivation of health utilities’; ‘Impact and cost-effectiveness analysis’ |
| Valuation of outcomes | 13 | Describe the population and methods used to measure and value outcomes. | ‘Model description’; ‘Derivation of health utilities’; ‘Impact and cost-effectiveness analysis’ |
| Measurement and valuation of resources and costs | 14 | Describe how costs were valued. | ‘Estimation of costs’; ‘Impact and cost-effectiveness analysis’; ‘Sensitivity analyses’; Tables 2 & 3; Supplementary Methods; Supplementary Tables S2 & S4 |
| Currency, price date, and conversion | 15 | Report the dates of the estimated resource quantities and unit costs, plus the currency and year of conversion. | ‘Estimation of costs’; ‘Sensitivity analyses’; Supplementary Methods; Supplementary Tables S2 & S4 |
| Rationale and description of model | 16 | If modelling is used, describe in detail and why used. Report if the model is publicly available and where it can be accessed. | ‘Model description’; Figure 1 |
| Analytics and assumptions | 17 | Describe any methods for analysing or statistically transforming data, any extrapolation methods, and approaches for validating any model used. | ‘Impact and cost-effectiveness analysis’; ‘Sensitivity analyses’; Supplementary Methods |
| Characterising heterogeneity | 18 | Describe any methods used for estimating how the results of the study vary for subgroups. | ‘Impact and cost-effectiveness analysis’; ‘Sensitivity analyses’; Supplementary Methods |
| Characterising distributional effects | 19 | Describe how impacts are distributed across different individuals or adjustments made to reflect priority populations. | ‘Model parameterisation’; Table 1; Supplementary Methods |
| Characterising uncertainty | 20 | Describe methods to characterise any sources of uncertainty in the analysis. | ‘Model parameterisation’; ‘Estimation of costs’; Derivation of health utilities’; ‘Impact and cost-effectiveness analysis’; ‘Sensitivity analyses’; Table 1; Supplementary Methods |
| Approach to engagement with patients and others affected by the study | 21 | Describe any approaches to engage patients or service recipients, the general public, communities, or stakeholders (such as clinicians or payers) in the design of the study. | Not applicable |
| **Results** |  |  |  |
| Study parameters | 22 | Report all analytic inputs (such as values, ranges, references) including uncertainty or distributional assumptions. | Tables 1-3; Supplementary Tables S1-S4 |
| Summary of main results | 23 | Report the mean values for the main categories of costs and outcomes of interest and summarise them in the most appropriate overall measure. | ‘Costing analysis’; ‘HRQoL analysis’; Cost of HCV treatment pathways’; ‘Health impact of treatment’; Cost-effectiveness of integrated treatment pathway’; Figures 2-5; Table 4; Supplementary Figures S1-S8; Supplementary Tables S5-S8 |
| Effect of uncertainty | 24 | Describe how uncertainty about analytic judgements, inputs, or projections affect findings. Report the effect of choice of discount rate and time horizon, if applicable. | ‘Costing analysis’; ‘HRQoL analysis’; Cost of HCV treatment pathways’; ‘Health impact of treatment’; Cost-effectiveness of integrated treatment pathway’; ‘Univariate sensitivity analysis’; ‘Variation in cost-effectiveness of integrated treatment across selected settings’; Figures 2-5; Table 4; Supplementary Figures S1-S8; Supplementary Tables S5-S8 |
| Effect of engagement with patients and others affected by the study | 25 | Report on any different patient/service recipient, general public, community, or stakeholder involvement made to the approach or findings of the study. | Not applicable; see also Discussion for wider impact |
| **Discussion** |  |  |  |
| Study findings, limitations, generalisability, and current knowledge | 26 | Report key findings, limitations, ethical or equity considerations not captured, and how these could affect patients, policy, or practice. | Discussion; ‘Strengths and limitations’; ‘Comparison with other studies’; ‘Conclusions and implications’ |
| Other relevant information |  |  |  |
| Source of funding | 27 | Describe how the study was funded and any role of the funder in the identification, design, conduct, and reporting of the analysis. | Role of the Funding Source statement |
| Conflicts of interest | 28 | Report authors conflicts of interest according to journal policy or International Committee of Medical Journal Editors requirements. | Declaration of Interests statement |

Supplementary References

1. Fadnes LT, Aas CF, Vold JH, Leiva RA, Ohldieck C, Chalabianloo F *et al.* Integrated treatment of hepatitis C virus infection among people who inject drugs: A multicenter randomized controlled trial (INTRO-HCV). *PLoS Med* 2021; 18: e1003653.
2. Kanwal F, Kramer JR, Ilyas J, Duan Z, El-Serag HB. HCV genotype 3 is associated with an increased risk of cirrhosis and hepatocellular cancer in a national sample of U.S. Veterans with HCV. *Hepatology* 2014; 60: 98–105.
3. Deans GD, Raffa JD, Lai C, Fischer B, Krajden M, Amin J *et al.* Mortality in a large community-based cohort of inner-city residents in Vancouver, Canada. *Cmaj Open* 2013; 1: E68–E76.
4. Hayashi K, Milloy M, Wood E, Dong H, Montaner JS, Kerr T. Predictors of liver‐related death among people who inject drugs in Vancouver, Canada: a 15‐year prospective cohort study. *J Int Aids Soc* 2014; 17: 19296.
5. Herdman M, Gudex C, Lloyd A, Janssen MF, Kind P, Parkin D *et al.* Development and preliminary testing of the new five-level version of EQ-5D (EQ-5D-5L). *Qual Life Res* 2011; 20: 1727–1736.
6. Buchanan-Hughes AM, Buti M, Hanman K, Langford B, Wright M, Eddowes LA. Health state utility values measured using the EuroQol 5-dimensions questionnaire in adults with chronic hepatitis C: a systematic literature review and meta-analysis. *Qual Life Res* 2019; 28: 297–319.
7. Stavem K, Augestad LA, Kristiansen IS, Rand K. General population norms for the EQ-5D-3L in Norway: comparison of postal and web surveys. *Health Qual Life Out* 2018; 16: 204.
8. Statens Legemiddelverk. Guidelines for the submission of documentation for single technology assessment (STA) of pharmaceuticals. .
9. Dolan P. Modeling Valuations for EuroQol Health States. *Med Care* 1997; 35: 1095–1108.
10. Hout B van, Janssen MF, Feng Y-S, Kohlmann T, Busschbach J, Golicki D *et al.* Interim Scoring for the EQ-5D-5L: Mapping the EQ-5D-5L to EQ-5D-3L Value Sets. *Value Health* 2012; 15: 708–715.
11. Ward Z, Mafirakureva N, Stone J, Keevans M, Betts-Symonds G, Crowley D *et al.* Cost-effectiveness of mass screening for Hepatitis C virus among all inmates in an Irish prison. *Int J Drug Policy* 2021; 96: 103394.
12. Erman A, Krahn MD, Hansen T, Wong J, Bielecki JM, Feld JJ *et al.* Estimation of fibrosis progression rates for chronic hepatitis C: a systematic review and meta-analysis update. *BMJ Open* 2019; 9: e027491.
13. Shepherd J, Jones J, Hartwell D, Davidson P, Price A, Waugh N. Interferon alfa (pegylated and non-pegylated) and ribavirin for the treatment of mild chronic hepatitis C: a systematic review and economic evaluation. *Health Technol Asses* 2007; 11: 1–205, iii.
14. Meer AJ van der, Veldt BJ, Feld JJ, Wedemeyer H, Dufour J-F, Lammert F *et al.* Association between sustained virological response and all-cause mortality among patients with chronic hepatitis C and advanced hepatic fibrosis. *JAMA* 2012; 308: 2584–2593.
15. Morgan RL, Baack B, Smith BD, Yartel A, Pitasi M, Falck-Ytter Y. Eradication of hepatitis C virus infection and the development of hepatocellular carcinoma: a meta-analysis of observational studies. *Ann Intern Med* 2013; 158: 329–337.
16. World Health Organization. INTERIM GUIDANCE FOR COUNTRY VALIDATION OF VIRAL HEPATITIS ELIMINATION - JUNE 2021. 2021; 1–96.
17. Caven M, Baiano CX, Robinson EM, Stephens B, Macpherson I, Dillon JF. Hepatitis C reinfection by treatment pathway among people who inject drugs in Tayside, Scotland. *J Viral Hepatitis* 2021; 28: 1744–1750.
18. Hartwell D, Jones J, Baxter L, Shepherd J. Peginterferon alfa and ribavirin for chronic hepatitis C in patients eligible for shortened treatment, re-treatment or in HCV/HIV co-infection: a systematic review and economic evaluation. *Health Technol Assess* 2011; 15: i-xii-1–210.
19. Ward Z, Reynolds R, Campbell L, Martin NK, Harrison G, Irving W *et al.* Cost‐effectiveness of the HepCATT intervention in specialist drug clinics to improve case‐finding and engagement with HCV treatment for people who inject drugs in England. *Addiction* 2020; 115: 1509–1521.
20. Gutkind S, Schackman BR, Morgan JR, Leff JA, Agyemang L, Murphy SM *et al.* Cost-effectiveness of Hepatitis C Virus Treatment Models for People Who Inject Drugs in Opioid Agonist Treatment Programs. *Clin Infect Dis* 2019; 70: 1397–1405.
21. Cousien A, Tran VC, Deuffic-Burban S, Jauffret-Roustide M, Mabileau G, Dhersin JS *et al.* Effectiveness and cost-effectiveness of interventions targeting harm reduction and chronic hepatitis C cascade of care in people who inject drugs: The case of France. *J Viral Hepat* 2018; 25: 1197–1207.
22. Scott N, Iser DM, Thompson AJ, Doyle JS, Hellard M. Cost-effectiveness of treating chronic hepatitis C virus with direct-acting antivirals in people who inject drugs in Australia. *J Gastroenterol Hepatol* 2016; 31: 872–882.
23. Scott N, Palmer A, Tidhar T, Stoove M, Sacks-Davis RS, Doyle JS *et al.* Assessment of the cost-effectiveness of Australia’s risk-sharing agreement for direct-acting antiviral treatments for hepatitis C: a modelling study. *Lancet Regional Heal - West Pac* 2022; 18: 100316.
24. Grishchenko M, Grieve RD, Sweeting MJ, Angelis DD, Thomson BJ, Ryder SD *et al.* Cost-effectiveness of pegylated interferon and ribavirin for patients with chronic hepatitis C treated in routine clinical practice. *Int J Technol Assess Health Care* 2009; 25: 171–180.
25. McAdam-Marx C, McGarry LJ, Hane CA, Biskupiak J, Deniz B, Brixner DI. All-Cause and Incremental Per Patient Per Year Cost Associated with Chronic Hepatitis C Virus and Associated Liver Complications in the United States: A Managed Care Perspective. *J Manag Care Pharm* 2011; 17: 531–546.
26. Chhatwal J, Kanwal F, Roberts MS, Dunn MA. Cost-Effectiveness and Budget Impact of Hepatitis C Virus Treatment With Sofosbuvir and Ledipasvir in the United States. *Ann Intern Med* 2015; 162: 397–406.
27. Schwarzinger M, Deuffic-Burban S, Mallet V, Pol S, Pageaux G-P, Canva-Delcambre V *et al.* 49 Lifetime costs attributable to chronic hepatitis C from the French healthcare perspective (ANRS N°12188). *J Hepatol* 2013; 58: S21–S22.
28. Statistisk sentralbyrå (Statistics Norway). Statistikkbanken (Statbank). <https://www.ssb.no.>
29. OECD Producer price indices (PPI) (indicator). <https://stats.oecd.org/Index.aspx?DataSetCode=MEI_PRICES_PPI#.>
30. US Bureau of Labor Statistics. <https://www.bls.gov/ppi/detailed-report/.>
31. Australian Bureau of Statistics. <https://www.abs.gov.au/statistics/economy/price-indexes-and-inflation/producer-price-indexes-australia/.>
32. Husereau D, Drummond M, Augustovski F, Bekker-Grob E de, Briggs AH, Carswell C *et al.* Consolidated Health Economic Evaluation Reporting Standards 2022 (CHEERS 2022) statement: updated reporting guidance for health economic evaluations. *Bmj* 2022; 376: e067975.
